# Measuring and modeling energy and power consumption in living microbial cells with a synthetic ATP reporter

**DOI:** 10.1186/s12915-021-01023-2

**Published:** 2021-05-17

**Authors:** Yijie Deng, Douglas Raymond Beahm, Steven Ionov, Rahul Sarpeshkar

**Affiliations:** 1 Thayer School of Engineering, Dartmouth College, Hanover, NH 03755 USA; 2 Departments of Engineering, Microbiology & Immunology, Physics, and Molecular and Systems Biology, Dartmouth College, Hanover, NH 03755 USA

**Keywords:** ATP biosensor, ATP dynamics, Metabolism, Cellular power consumption, Cell energetics, Bacterial kinetics, Kinetic circuit models

## Abstract

**Background:**

Adenosine triphosphate (ATP) is the main energy carrier in living organisms, critical for metabolism and essential physiological processes. In humans, abnormal regulation of energy levels (ATP concentration) and power consumption (ATP consumption flux) in cells is associated with numerous diseases from cancer, to viral infection and immune dysfunction, while in microbes it influences their responses to drugs and other stresses. The measurement and modeling of ATP dynamics in cells is therefore a critical component in understanding fundamental physiology and its role in pathology. Despite the importance of ATP, our current understanding of energy dynamics and homeostasis in living cells has been limited by the lack of easy-to-use ATP sensors and the lack of models that enable accurate estimates of energy and power consumption related to these ATP dynamics. Here we describe a dynamic model and an ATP reporter that tracks ATP in *E. coli* over different growth phases.

**Results:**

The reporter is made by fusing an ATP-sensing *rrnB* P1 promoter with a fast-folding and fast-degrading GFP. Good correlations between reporter GFP and cellular ATP were obtained in *E. coli* growing in both minimal and rich media and in various strains. The ATP reporter can reliably monitor bacterial ATP dynamics in response to nutrient availability. Fitting the dynamics of experimental data corresponding to cell growth, glucose, acetate, dissolved oxygen, and ATP yielded a mathematical and circuit model. This model can accurately predict cellular energy and power consumption under various conditions. We found that cellular power consumption varies significantly from approximately 0.8 and 0.2 million ATP/s for a tested strain during lag and stationary phases to 6.4 million ATP/s during exponential phase, indicating ~ 8–30-fold changes of metabolic rates among different growth phases. Bacteria turn over their cellular ATP pool a few times per second during the exponential phase and slow this rate by ~ 2–5-fold in lag and stationary phases.

**Conclusion:**

Our *rrnB* P1-GFP reporter and kinetic circuit model provide a fast and simple way to monitor and predict energy and power consumption dynamics in bacterial cells, which can impact fundamental scientific studies and applied medical treatments in the future.

## Background

Adenosine triphosphate (ATP) is the key energy source for all living organisms, essential to fundamental processes in all cells from metabolism to DNA replication and protein synthesis [1]. In humans, abnormal cellular ATP levels and power consumption (ATP consumption rate), as can be determined by measuring and modeling ATP, are related to many diseases, such as cancer, aging, obesity, diabetes, neuronal disorders, viral infections, and immune dysfunctions [1–6]. In bacteria, ATP dynamics are directly related to bacterial metabolic activity, physiology, and behaviors under varying conditions and stresses [7–9]. For example, low ATP levels contribute to bacterial resistance/persistence in response to antibiotic treatments [9–12]. Despite ATP’s importance, our current understanding of ATP dynamics and homeostasis in cells has been limited by the lack of readily available and easy-to-use continuous ATP biosensors as well as by the shortage of accurate dynamic models to determine ATP fluxes.

The quantitative and continuous measurement of cellular ATP has proven challenging. Conventional methods, such as luciferase assays, require efficient lysis of cells and thus preclude real-time and continuous intracellular ATP measurements [13]. To this end, several genetically encoded ATP biosensors have been developed, such as the fluorescence resonance energy transfer FRET-based ATeam biosensor [14], the bioluminescence resonance energy transfer BRET-based BTeam biosensor [13], and the new ATeam3.10 biosensor [15]. These ratio-metric biosensors measure ATP, irrespective of their expression levels in the cell, and function well in slow-growing mammalian cell lines. To monitor cellular ATP in fast-growing bacteria, Yaginuma et al. developed a QUEEN ATP sensor but wider applications of this sensor in bacteria have not been reported, possibly due to its relatively dim signal and sensitivity to temperature [16]. Furthermore, these biosensors require expensive fluorescence microscopes and time-consuming procedures for sample preparation and image analysis. These limitations make it challenging to continuously monitor intracellular ATP, e.g., in synthetic biological applications in the body that require fast, cheap, and continuous sensing of ATP in living microbial or other cells. Monitoring such ATP dynamics can predict nutrients, cellular stresses, disease states, or efficacy of drug treatments [2–7, 9, 11] and might be used to modulate or actuate therapeutic molecular release in response to cellular energetics.

Given that protein synthesis is the major energy-consuming process in the cell, ribosome synthesis must be tightly controlled by ATP/GTP availability in order to maintain ATP homeostasis [17–20]. The activity of a ribosomal RNA (rRNA) promoter, *rrnB* P1, has been shown to depend on cellular ATP level in *E. coli* [17, 18]. Upon binding, an RNA polymerase holoenzyme (RNAP) and the *rrnB* P1 promoter form a very short-lived open complex; this unstable open complex requires an unusually high concentration of ATP (Kd in the mM range) to initiate the transcription of rRNA [17, 18, 21, 22]. The sensitivity of the *rrnB* P1 promoter to ATP is attributed to its specific features, including non-consensus -35 hexamers, non-optimal spacing between -35 and -10 hexamers, and a GC- rich discriminator [23, 24]. The requirement of high ATP concentration for transcription initiation is the rate-limiting step and allows for the regulation of rRNA production by changing ATP levels as long as they are not saturating [23, 24]. Therefore, the activity of the *rrnB* P1 promoter was proposed as a sensitive ATP indicator in *E. coli* [10, 17, 18]. However, systematic and quantitative analyses of *rrnB* P1-based ATP reporters that enable dynamic energy and power consumption measurements have thus far been missing. The combinatorial use of such ATP reporter and dynamic models could enable efficient determination of cellular energetics across various growth phases.

In this work, we designed and screened a series of synthetic ATP reporters in *E. coli*. The ATP reporters were made by fusing the ATP-sensing *rrnB* P1 promoter with the gene of a fast-folding GFP (GFP-mut2) that folds within minutes [25]. An SsrA protease degradation tag [26, 27] fused to the C-terminus of the GFP also enabled its rapid degradation. Thus, the GFP produced from the *rrnB* P1 promoter in response to cellular ATP enabled relatively fast tracking of ATP in *E. coli.* We tested the performance of the reporter in minimal and rich media. Even though the activity of the *rrnB* P1 promoter is also affected by high levels of guanosine tetraphosphate (ppGpp) under starvation conditions [18, 20], we found that our ATP reporter can faithfully track cellular ATP levels under different experimental conditions regardless of potential ppGpp presence. After verifying the performance of the *rrnB* P1-based ATP reporter in various media and *E. coli* strains, we utilized it to study how bacterial ATP dynamics change in response to varying nutrients, including glucose and phosphate. To demonstrate the accuracy of the ATP reporter in power consumption measurements during bacterial growth, we developed a kinetic model and an electrical circuit model for bacterial growth in minimal medium. Our ATP reporter measurements and model enable us to quantitatively estimate intracellular ATP power consumption (ATP flux) in living cells, which is hard to estimate by luciferase-based or other ATP sensors. We show that our work can help quantify striking changes in ATP dynamics and power consumption across bacterial growth phases.

## Results

### Comparison of different *rrnB* P1-GFP reporter constructs

Our reporter consists of an *rrnB* P1 promoter fused to a fast-folding and fast-degrading GFP that is composed of GFP-mut2 [25] and an SsrA-tag. We made four versions of our ATP reporter including two on a low-copy plasmid (LC) and two on a high copy plasmid (HC) with varying strengths of ribosomal binding sequences (RBS). We evaluated the response of the reporters during growth in the EZ-rich medium and the MOPS minimal medium. Once bacteria are seeded in a fresh medium, abundant nutrients allow cells to accumulate ATP, which reaches a constant level or a steady state during the exponential phase [28–30]; ATP then falls in stationary phase when nutrients become depleted. Therefore, a good ATP reporter is expected to have GFP dynamics similar to cellular ATP dynamics and display bell-shaped or non-monotonic characteristics that are well correlated with nutrient availability and growth phase.

**Fig. 1:**
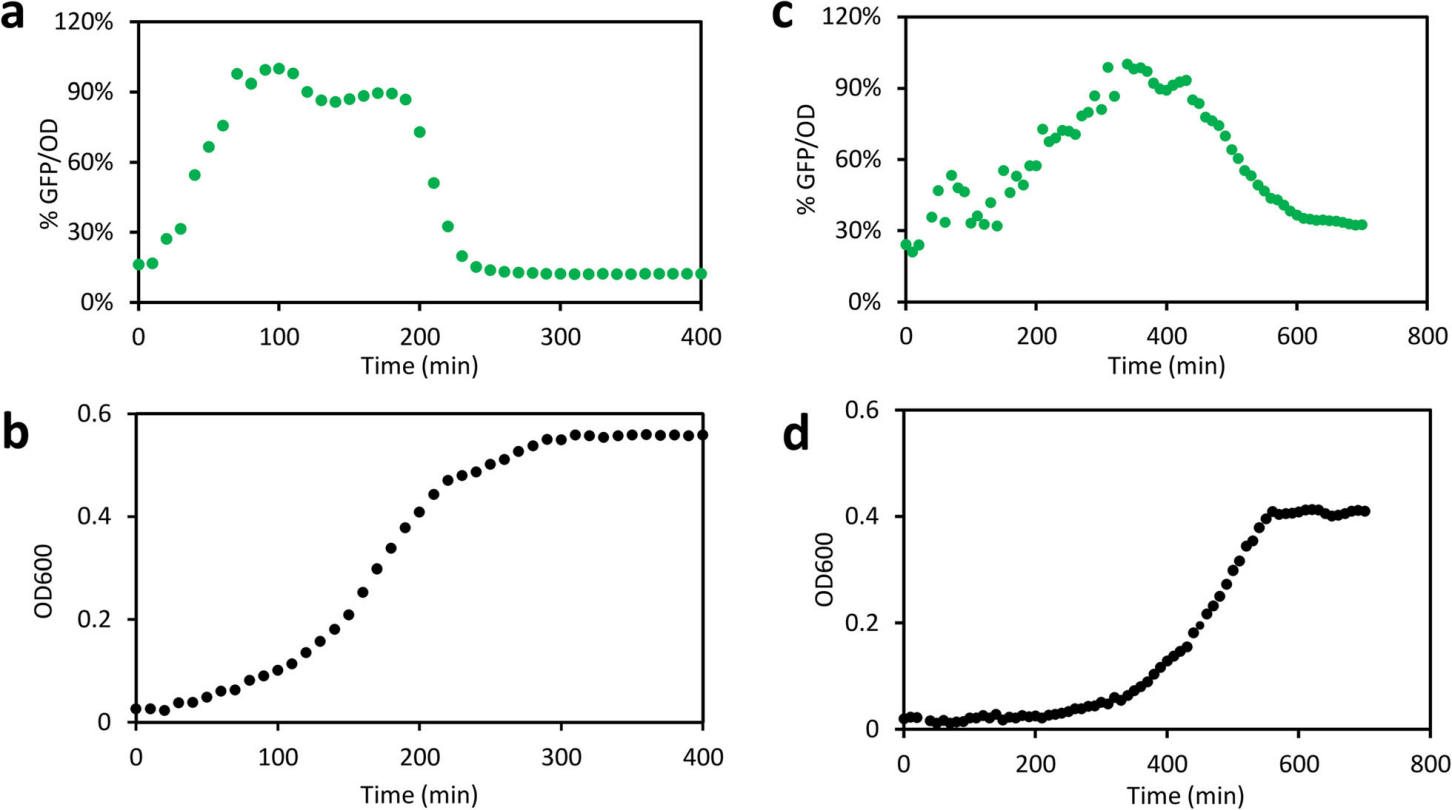
Fluorescence and growth characteristics of HC-M ATP reporter in rich and minimal media. **a**, **b** Normalized cellular GFP dynamics [% (GFP/OD)] (**a**) and growth (**b**) of *E. coli* carrying the HC-M ATP reporter grown in the rich medium. **c**, **d** Normalized cellular GFP dynamics [% (GFP/OD)] (**c**) and growth (**d**) of *E. coli* carrying the HC-M ATP reporter grown in minimal medium. The *E. coli* NEB 10-beta strain with the HC-M ATP reporter was grown in EZ rich medium with 5 mM glucose or MOPS minimal medium with 10 mM glucose. Bacteria were grown in black 96-well plates with shaking. GFP (ex485/em528) and OD600 were measured with a microplate reader (Molecular Devices, Inc.). The cellular GFP signals, GFP/OD, were normalized by their own peak GFP/OD values (100%). Each data point is the mean value of at least three independent experiments. The standard deviations were small (< 15% of the mean) and not shown

We compared GFP signals of four versions of the ATP reporter and one control construct without an ATP-sensitive promoter (Additional file 1: Figure S1). The high-copy-plasmid ATP reporter with a relatively low RBS strength, denoted HC-M, displayed the expected bell-shaped dynamics in both minimal and rich media (Fig. 1). It had low fluorescence signals during lag and stationary phases and a plateau during the exponential phase. Another ATP reporter (HC-E) displayed similar characteristics but did not correlate well with ATP in a further test (Additional file 2: Figure S2). Therefore, we used the HC-M ATP reporter for all further studies in this work.

### Correlation of the *rrnB* P1-GFP reporter measurements with ATP levels in *E. coli*

**Fig. 2:**
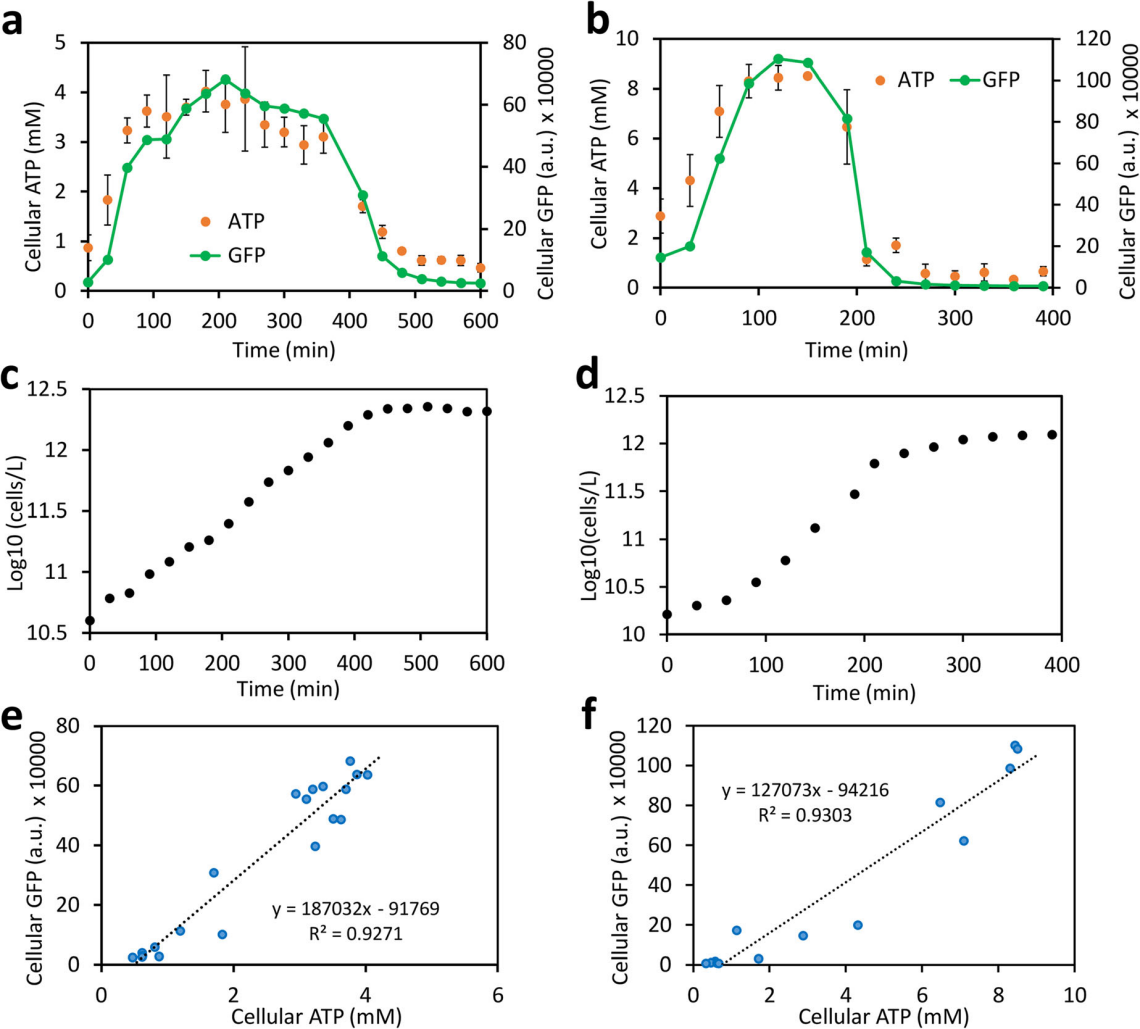
Correlation between the *rrnB* P1-GFP reporter and ATP. **a**, **b** Dynamics of the GFP reporter and cellular ATP levels in *E. coli* in minimal (**a**) and EZ-rich medium (**b**). Cellular GFP fluorescence for each sample (arbitrary fluorescence unit, a.u.) was measured by flow cytometry. Cellular ATP levels were determined by a standard luciferase assay and converted to cellular concentration in mM. Growth of bacteria is shown in minimal medium (**c**) and in rich medium (**d**). Bacterial cell counts were estimated by flow cytometry corrected with counting beads. **e**, **f** Linear correlations between cellular ATP concentration and cellular GFP levels in minimal (**e**) and rich medium (**f**). All experiments used the same BW25113 strain for consistency. All data points are mean values of at least three independent biological replicates with one standard deviation (SD). The SD for growth and the GFP signal were relatively small (< 15%) and are not shown

We next evaluated the ATP-tracking performance of the HC-M reporter in *E. coli* grown in the minimal medium and the rich medium. The cellular GFP signals during growth at different time points were measured by flow cytometry; ATP in each culture sample was measured using a commercial luciferase assay and converted to cellular concentration as described in the “Methods” section. We found that the HC-M reporter tracked cellular ATP faithfully over the lag, exponential, and stationary growth phases in both the minimal medium and rich medium (Fig. 2a,b). Both GFP and ATP levels rose quickly in cells after seeding in the fresh medium and then remained at a steady state during the exponential phase, followed by a rapid drop of both signals to a basal level in the stationary phase (Fig. 2a–d). Good linear correlations were observed between cellular ATP and GFP values for both the minimal medium (*R*^2^ = 0.9271) and the rich medium (*R*^2^ = 0.9303) (Fig. 2e, f). Our results validate that the HC-M ATP reporter is able to track cellular ATP faithfully across different growth phases.

Flow cytometry analysis shows that bacterial population was uniform across growth phases in both the rich and minimal media, except that a slight heterogeneity was noticed during the transition time between the log and stationary phases (Additional files 3, 4: Figures S3, S4), which might be due to the stochastic, heterogeneous, and asynchronous nature of bacterial population during growth [31–33]. However, this temporal heterogeneity was minimal and does not affect the performance of the HC-M reporter to measure cellular ATP, given that the mean GFP levels of the population still correlate well with the cellular ATP levels (Fig. 2). Overall, we found that the HC-M correlates closely with the changes of cellular ATP and is a reliable tool to estimate cellular ATP over bacterial growth phases.

**Fig. 3:**
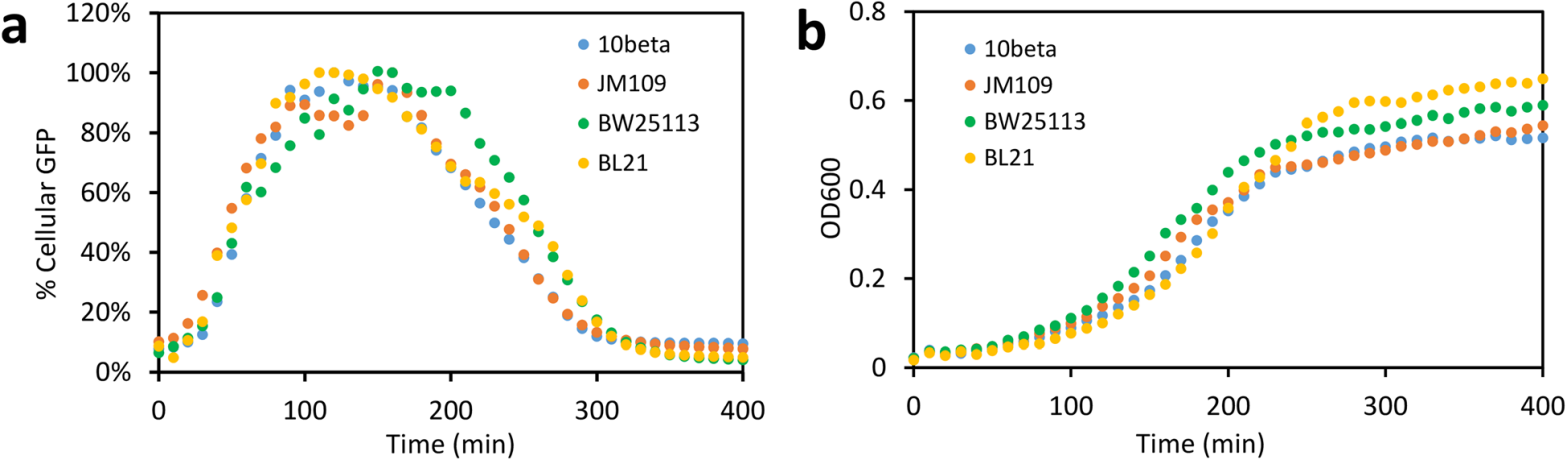
Robustness of the *rrnB* P1 ATP reporter in different *E. coli* strains. **a** Dynamics of cellular GFP (GFP/OD) of four *E. coli* strains (NEB 10-beta, BW25113, JM109DE3, and BL21DE3) with HC-M ATP reporter in rich medium with 6 mM glucose. **b** Corresponding bacterial growth among the four strains in the same medium. Bacterial strains were grown in black 96-well plates and GFP (ex485/em528) and OD600 were measured by a microplate reader. Cellular GFP (GFP/OD) signals of each strain were normalized to their respective peak GFP/OD values (100%) for comparison among different strains. Each data point represents the mean of at least three independent replicates with a standard deviation < 13% of the mean

Our ATP reporter also works in different *E. coli* strains. Besides the BW25113 strain we tested above (Fig. 2), three other strains including JM109(DE3), NEB-10beta, and BL21(DE3) showed very similar cellular GFP dynamics (Fig. 3) even though they have significantly different genetic backgrounds. These results indicate that the reporter is robust and can track ATP in a broad range of *E. coli* strains. For example, we also verified that the reporter GFP signals correlate well with cellular ATP in JM109(DE3) strain grown in rich medium (Additional file 5: Figure S5a,c) and in BL21(DE3) strain grown in minimal medium (Additional file 5: Figure S5b,d). As in any accurate sensing scheme, for the best quantitative accuracy, calibration curves for a given strain in typical culture conditions will need to be established to compensate for strain-to-strain and media variations.

### Monitoring ATP dynamics in bacteria grown with different limiting nutrients

**Fig. 4:**
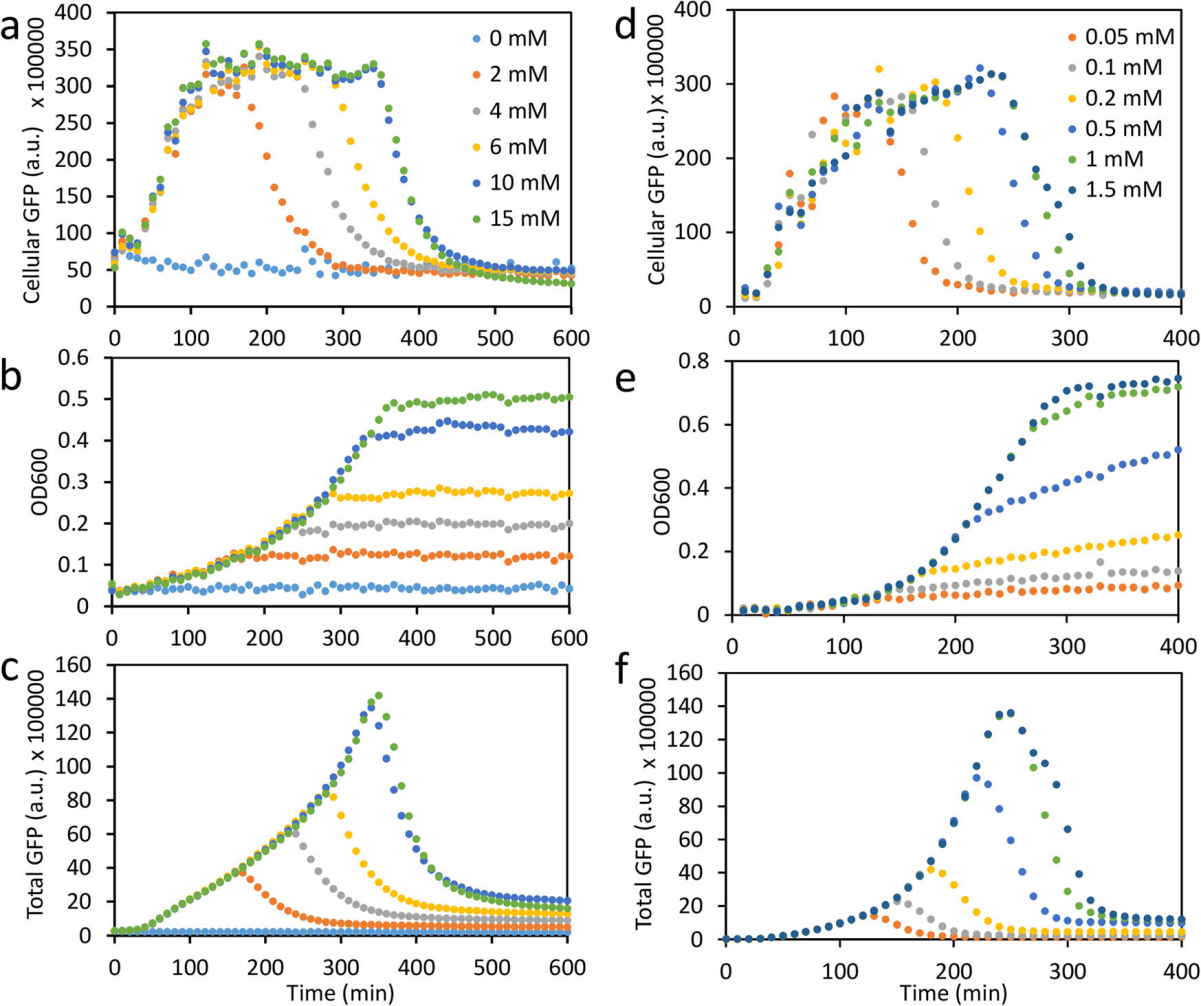
Cellular GFP (ATP) dynamics in response to rate-limiting nutrients using our ATP-dependent reporter. **a**–**c** Cellular GFP dynamics (**a**), bacterial growth (**b**), and total-population-level GFP dynamics (**c**), in response to different amounts of glucose (0–15 mM) in minimal medium. As in Fig. 2, the cellular GFP serves as a good proxy for cellular ATP and helps us monitor it in real time. **d**–**f** Cellular GFP dynamics (**d**), bacterial growth (**e**), and total-population-level GFP dynamics (**f**) in response to different amounts of phosphate (0.05–1.5 mM) in rich medium. Each data point represents the mean of four independent biological replicates with standard deviation < 15% of the mean. Bacteria were grown in black 96-well plates. GFP (ex485/em528) and OD600 were measured by a microplate reader. Cellular GFP is defined as the total GFP divided by OD

Energy dynamics in bacteria are essential to their metabolic activities and phenotypes under different conditions, including their persistence to stresses [7, 8, 10, 34, 35]. Thus, our ATP reporter could be useful in studying ATP dynamics in response to stresses such as limiting nutrients or drug treatments. For example, we employed our reporter to monitor bacterial ATP dynamics in response to glucose. Glucose is the sole energy source in the minimal medium and thus applying more of the limiting-nutrient, glucose (0 to 10 mM), to bacteria in this medium can sustain a longer exponential phase and result in a longer period of steady-state ATP levels. When glucose is no longer limiting (e.g., changing it from 10 to 15 mM in Fig. 4a, b), there is little effect by glucose on ATP dynamics. Figure 4c shows that the total GFP of the bacterial population accumulates with growth and then decays after all the glucose in the medium has been consumed. Overall, Fig. 4a–c show that cellular ATP dynamics show a good correlation with bacterial growth, glucose consumption, and glucose availability. In another example, our reporter also reliably tracks bacterial ATP dynamics in response to phosphate availability (Fig. 4d–f). Measuring ATP dynamics in response to nutrient availability and stresses can provide deeper insight into the underlying bacterial metabolism, which is difficult to obtain by just monitoring growth curves.

### Kinetic model development for the measurement of ATP dynamics

**Fig. 5:**
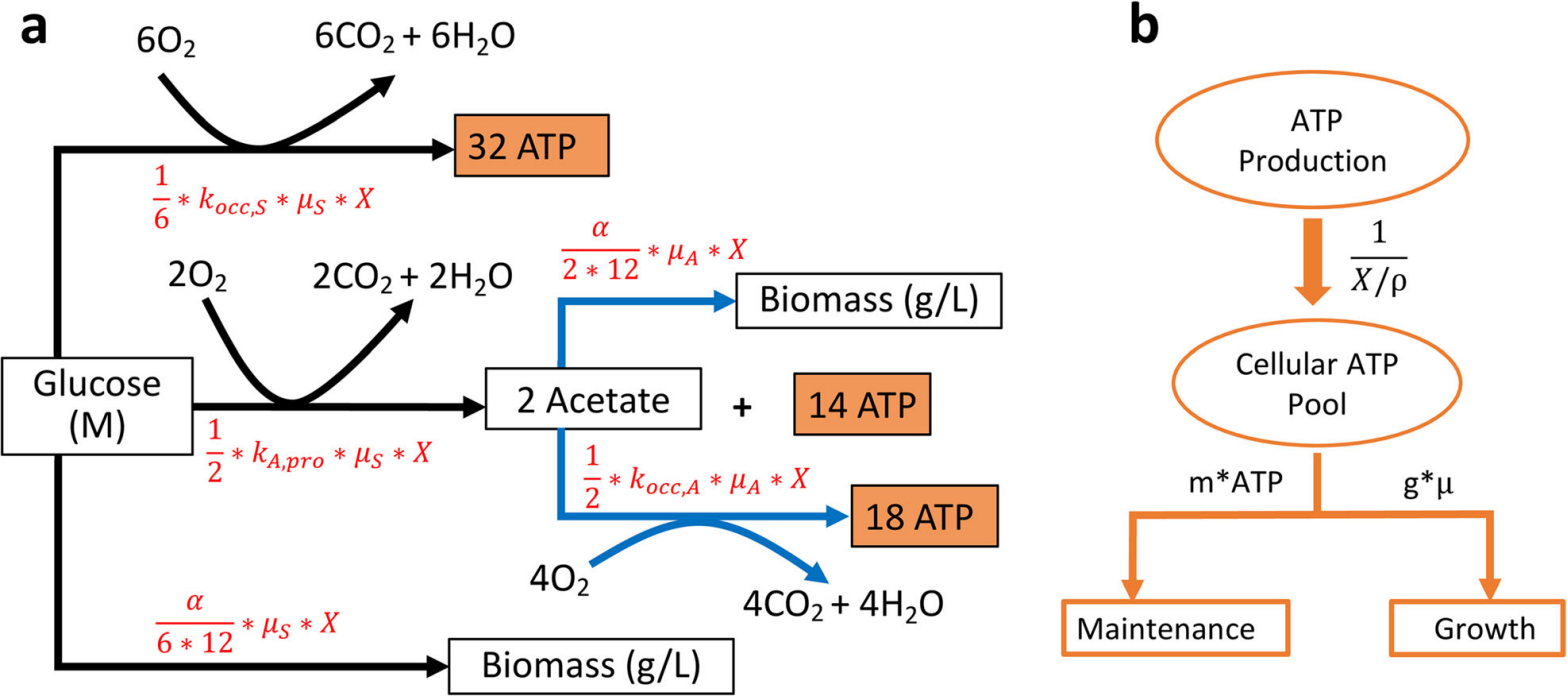
Workflow of kinetic model development. **a** Black arrows correspond to glucose fluxes converting glucose to biomass, carbon dioxide and water (respiration), or to the byproduct acetate, the excretion flux. Similarly, blue arrows correspond to acetate fluxes converting acetate to biomass or to carbon dioxide and water. The stoichiometry is based on general biochemical processes for glucose and acetate metabolism. Acetate utilization only occurs after glucose is depleted. The mathematical terms on each arrow describe the flux, or consumption rate, for that path in (M/s). Given the glucose or acetate consumption rate for each pathway, the total ATP production rate can then be calculated via stoichiometry (mole product per mole substrate consumed). Biomass production is based on the carbon balance between the substrate consumed and cellular carbon produced (more details are provided in the “Methods” section). **b** All ATP production fluxes are gathered and converted to the cellular ATP pool. This pool is simultaneously drained for bacterial growth and for cell maintenance via the mathematical flux rate terms (M/s) as indicated in the figure

**Fig. 6:**
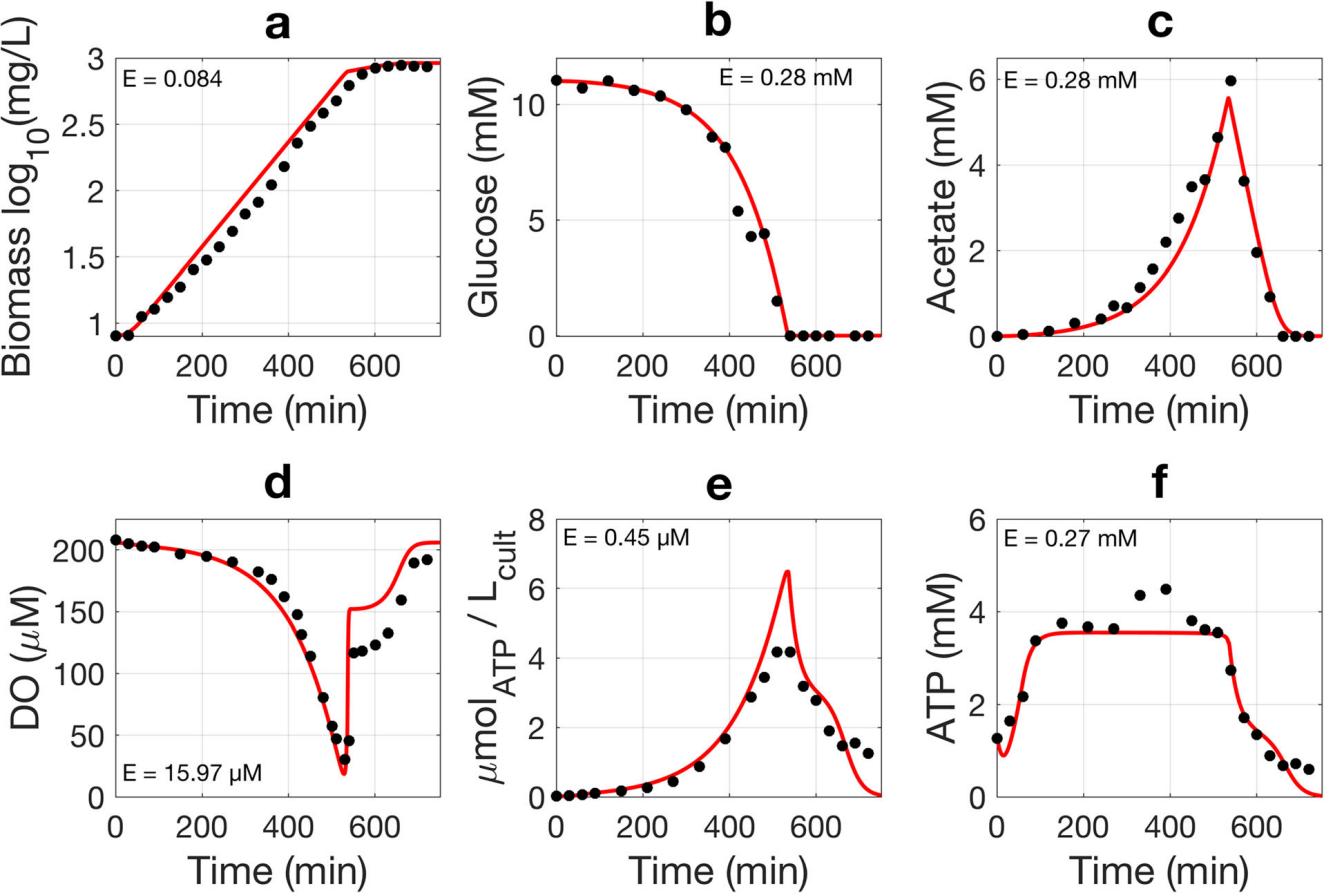
Experimental data and model simulation for batch-culture kinetics. The BW25113 strain with the ATP reporter was grown in minimal medium. Black circles represent measured experimental data (means of three independent replicates) and red lines represent model simulation results. Biomass (**a**) was estimated by multiplying the mass of a single cell with cell counts determined via flow cytometry. Glucose (**b**), acetate (**c**), and dissolved oxygen (**d**) were measured as described in the “Methods” section. ATP was measured by a standard luciferase assay. Population ATP (**e**) is indicated by the amount of ATP per L of culture. Cellular ATP (**f**) is calculated from the population ATP, cell counts, and the volume of a single cell. E is the average absolute error of the model in the units of the corresponding *y*-axis and indicates the model’s goodness of fit for each data fitting. The small E values indicate good fits of our model

We next sought to explore whether our ATP reporter can quantitatively estimate ATP dynamics and power consumption in bacteria. We developed a kinetic model and employed it with our ATP reporter to study growth kinetics and estimate power consumption during bacterial growth. Although cellular ATP can be directly estimated by our ATP reporter under similar conditions using the pre-established calibration curves (e.g., Fig. 2), determining energy fluxes or power consumption requires kinetic models with differential equations and the classic Monod equation [36], described in the “Methods” section in depth. The workflow is described in Fig. 5: The model includes glucose consumption, bacterial growth, acetate production corresponding to overflow metabolism and its subsequent consumption, oxygen dynamics, total population ATP dynamics, and individual cellular ATP dynamics. The differential equations can be conveniently represented and visualized as electric circuits as well (Additional file 6: Figure S6), as reported in our previous publications [37–43]. Our circuit models were simulated in the Cadence Virtuoso Analog Design Environment (Cadence Design Systems, Inc.). Cellular ATP dynamics can be determined by conversion from population-scale metabolic rates to single-cell values using cell counts and estimated cell volumes. Through these methods, ATP fluxes (global power consumption) and cellular power consumption (ATP/cell/s) during bacterial growth can be quantified. Our model was validated by experimental data fitting (Fig. 6), known physical constants for energy metabolism (e.g., in Fig. 5), and by sweep-sensitivity analyses, architected by varying initial glucose levels and growth rates (Additional file 7: Figure S7).

Bacteria were grown in batch cultures in minimal medium for easy analysis and modeling. Glucose was used to produce biomass and acetate and for aerobic respiration. Our model describes characteristic bacterial growth, glucose consumption, acetate production and consumption, and dissolved oxygen dynamics (Fig. 6). As Fig. 6 shows, once inoculated into the fresh medium, bacteria enter exponential phase quickly. Bacterial growth significantly slows when glucose is depleted but continues for a little while by consuming previously secreted acetate until acetate is also depleted (Fig. 6a–c). Aerobically growing *E. coli* generally produce acetate despite the presence of oxygen, a phenomenon known as the overflow metabolism [44, 45]. Dissolved oxygen (DO) levels remain relatively constant at the beginning of growth, in agreement with glucose dynamics (Fig. 6b, d). Oxygen consumption then increases dramatically on a population level during the exponential phase, resulting in a rapid drop in DO until glucose is depleted. DO then rises quickly to a level that supports slower acetate metabolism and slower growth. At the end of the experiment, when growth stops, DO levels return to steady-state lag values (Fig. 6a, d).

We then used our kinetic model to study ATP dynamics, which are determined by summing ATP production fluxes and subtracting ATP consumption fluxes (Fig. 5 and Figure S6), described in Eqs. 7–15. Population ATP levels, as expected, follow growth contours closely, peaking at the end of exponential phase and decreasing to a basal level during the stationary phase (Fig. 6e). Cellular ATP dynamics predicted by the model track luciferase measurements: ATP rises quickly and remains at the steady state during the exponential phase, followed by a fall upon glucose exhaustion (Fig. 6f).

The robustness of our model was further validated by two sensitivity analyses (Additional file 7: Figure S7): We varied inputs corresponding to initial glucose concentration and to the maximum specific growth rate, both of which are known to influence ATP dynamics, while keeping all other parameters constant. As expected, increasing glucose causes higher bacterial growth and an exponential phase of increased duration; peak population ATP also increases as more glucose is added (Additional file 7: Figure S7a-c). Most importantly, in strong agreement with our measured biological data from Fig. 4, the steady-state cellular ATP in the exponential phase is invariant with initial glucose concentration (Additional file 7: Figure S7c). Similarly, our model accurately predicts that glucose is depleted faster as growth rate increases, with different growth rates corresponding to different slopes in the growth curves (Additional file 7: Figure S7d-f). The cellular ATP in the exponential phase is also constant at different growth rates (Additional file 7: Figure S7e), in accord with previous reports that *E. coli* contain similar amounts of ATP at different steady-state growth rates [46, 47]. To match these previous reports, ATP homeostasis in our model requires only a slight increase of a single parameter, the growth-associated energy consumption coefficient, *g*, in Eq. 14. It is likely that ATP homeostasis in actual biological cells is more complex and bacteria at a higher growth rate might consume ATP faster, corresponding to a greater *g*. In sum, our relatively coarse-grained model still accounts for important input-output characteristics accurately.

The results of Fig. 6 and our sensitivity analyses suggest that our model can accurately capture the dynamics of biomass, glucose, acetate, oxygen, and ATP. It is thus useful for extracting ATP fluxes (power consumption) in *E. coli*, as we now discuss.

### Determining energy dynamics and power consumption by kinetic modeling

**Fig. 7:**
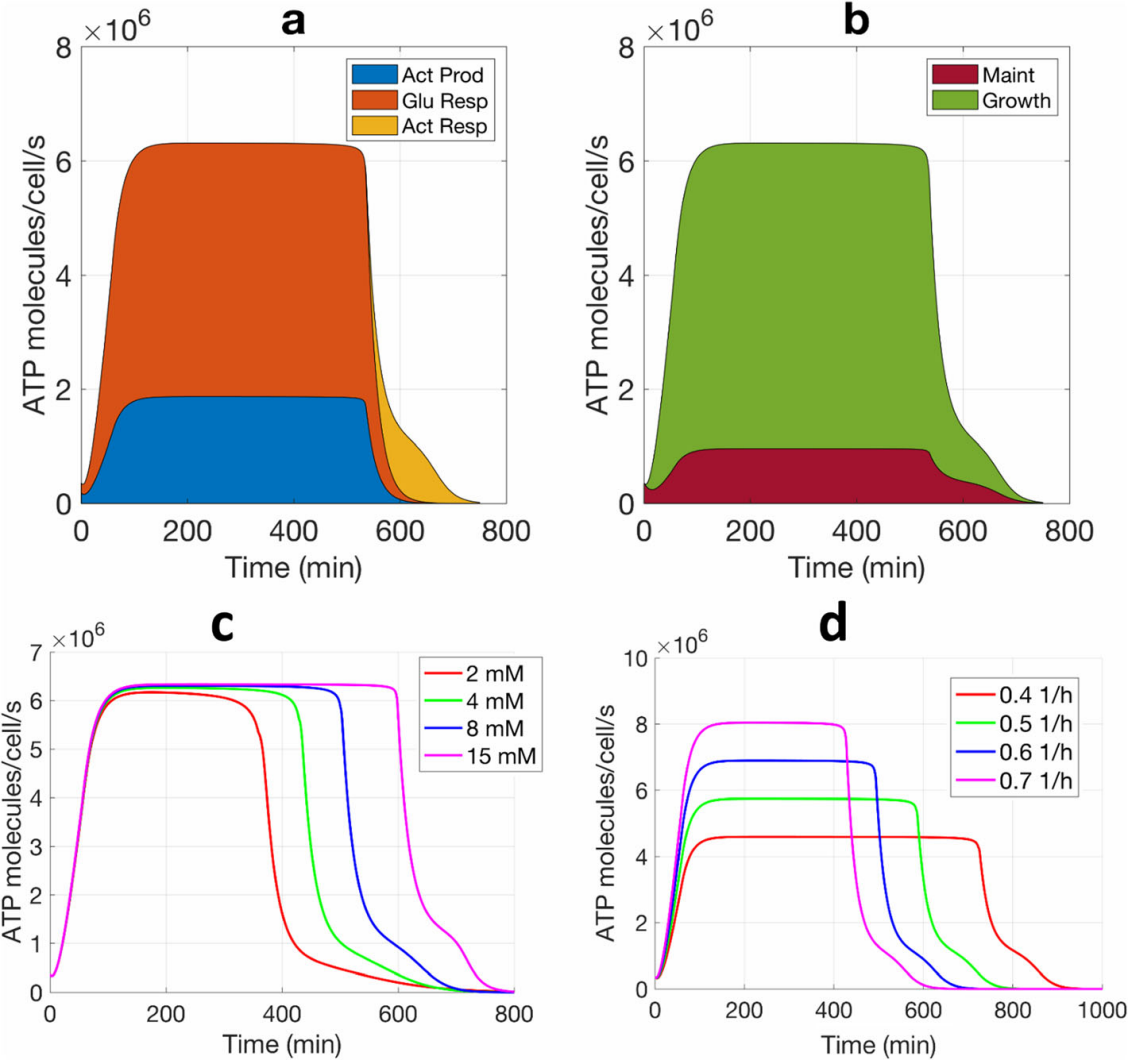
Determination of ATP fluxes by kinetic models that are derived by fitting measured experimental data. **a** Cellular ATP production fluxes from glucose aerobic respiration (Glu Resp), acetate production (Act Prod), and Act respiration (Act Resp). **b** ATP or power consumption fluxes for growth and cell maintenance (Maint.). The thickness of each colored zone indicates the contribution of each ATP flux and all colored zones sum up to the total ATP flux. The modeling used the same data and parameters from Fig. 6. **c** Model prediction of ATP consumption rates as initial glucose increases. **d** Model prediction of ATP consumption rates as growth rate increases. Initial glucose concentration is kept the same when varying the growth rate and vice versa

After validating our kinetic model with measured biological data in Fig. 6 and extracting model parameters (shown in Additional file 8: Table S1), we used the model to determine ATP production (based on glucose input) and power consumption (based on ATP consumption fluxes). Equations 7–15 (in the “Methods” section) capture the mathematics behind our model while Fig. 7a–d show some important results based on the model. The results show a total ATP production rate of approximately 6.4 million ATP/cell/s in exponential phase. Aerobic respiration contributes to over 72% of the total ATP production during exponential growth, first with glucose metabolism, and then with acetate metabolism (Fig. 7a). Acetate production also yields a considerable amount of ATP production when glucose is available. Notably, our model shows that ATP production rates correlate closely with oxygen consumption rates (Additional file 9: Figure S8), consistent with the idea that aerobic respiration is the major energy source. During the exponential phase, the ATP yield per O_2_ is estimated to be 5.74.

While aerobic respiration is the major power source, cellular growth consumes the most ATP during active growth. In our model, as shown in Fig. 5, ATP is consumed for bacterial growth as well as for cellular maintenance, e.g., for repairing cell membranes and damaged proteins and other cost-of-living functions [48–50]. During a short lag phase, the maintenance energy consumption accounts for a relatively large portion of ATP cost for bacteria, while during the exponential phase, growth accounts for the major ATP consumption (~ 85%) (Fig. 7b).

ATP consumption occurs simultaneously with ATP production with a near-perfect balance of the two fluxes, implying near-perfect ATP homeostasis and invariance of ATP’s steady-state concentration. Our model predicted that ATP production rate nearly equals its consumption rate so as to maintain the ATP homeostasis in cells (Fig. 7a, b), a result that is both experimentally observed and also critical in bacteria due to the high ATP turnover rate [8, 51, 52]. During the lag phase (0–60 min), the production and consumption rates of ATP both increase from 0.4 to 4.0 million ATP/cell/s. During the exponential phase, both rates increase rapidly to a steady-state value of 6.4 million ATP/cell/s, which is consistent with a previous report that growing *E. coli* consumes ATP at a rate between 1 and 10 million ATP/cell/s [51]. ATP fluxes fall to less than 0.5 million ATP/cell/s in the stationary phase.

Our model also correctly predicts that the cellular ATP consumption rate is independent of the initial glucose concentration, as shown in Fig. 7c: Even if the initial glucose concentration increases, the cellular ATP consumption rate remains at a homeostatically similar value in the exponential phase (steady-state ATP in Fig. 7c). However, since higher initial glucose concentration enables glucose depletion and consequent bacterial entry into stationary phase to occur later, it does lead to a longer exponential phase in Fig. 7c. In contrast, given that bacterial growth dominates power consumption, the cellular ATP consumption rate increases as growth rate increases in Fig. 7d. Here, an increased rate of glucose depletion with a higher growth rate leads to a shorter exponential phase. In another test, we changed the initial concentration of cellular ATP and found that it has minimal effect on the overall ATP dynamics and consumption rates (Additional file 10: Figure S9). Small dips in ATP dynamics were noticed, which are due to the transient imbalances in fluxes in the beginning of the lag phase, but by the time homeostatic equilibrium is reached at the beginning of the exponential phase, they do not affect the overall ATP dynamics and consumption rates. More complex models of ATP homeostasis with more details of phase and cell state can likely provide further improvements over our relatively simple model. Nevertheless, as Figs. 6, 7, and Figure S9 (Additional file 10) show, many aspects of measured biological data are fitted and captured.

Our model also shows that bacterial cells are incredibly energy-efficient. Given the consumption rate of 6.38 million ATP/s/cell and the free energy of 54 kJ/mole released from ATP under physiological conditions in *E. coli* [53, 54], the power consumption for exponential growth is computed to be 0.57 pW or 5.7 × 10^−13^ J/s (Table 1), consistent with the mean value of 0.49 pW reported previously in a prokaryotic cell [51]. Cellular ATP consumption rates during the lag phase average to about 0.82 million ATP/cell/s (Fig. 7b) and during the stationary phase to about 0.23 million ATP/cell/s, approximately 7.8-fold and 28-fold lower power consumptions than in the exponential phase (Table 1, Fig. 7).

**Table 1:** Calculated ATP dynamic values in *E. coli* BW25113 grown in minimal medium

	Lag phase		Exponential phase		Stationary phase	
	Luc	Reporter	Luc	Reporter	Luc	Reporter
Averaged ATP (mM)	0.86 ± 0.16	0.72 ± 0.12	3.50 ± 0.45	3.32 ± 0.28	0.58 ± 0.08	0.68 ± 0.07
ATP consumption rate (million ATP/cell/s)	0.82 ± 0.07	0.77 ± 0.11	6.38 ± 0.06	6.38 ± 0.06	0.23 ± 0.06	0.23 ± 0.06
Power consumption (pW)^a^	0.073 ± 0.006	0.069 ± 0.01	0.57 ± 0.01	0.57 ± 0.005	0.021 ± 0.005	0.021 ± 0.005
ATP turnover time (s)	0.64 ± 0.12	0.56 ± 0.09	0.33 ± 0.04	0.31 ± 0.03	1.5 ± 0.21	1.8 ± 0.18

^a^: power consumption was estimated from 54 kJ/mole ATP [53, 54]. All values were estimated from three independent biological experiments evaluated by the kinetic model. Luc indicates the calculated ATP data based on the luciferase assay while Reporter indicates ATP data based on the measurements from our GFP reporter

Given a cellular ATP concentration averaged at around 3.5 mM, we calculated the turnover time for ATP to be 0.33 s during the exponential phase. As expected, the ATP turnover rate is significantly slower in the lag phase (0.64 s) and stationary phase (1.5 s) than in the exponential phase (Table 1). The slower ATP turnover rate indicates a slowing of metabolism in bacteria adapting to a new growth condition during the lag phase or lacking nutrients during the stationary phase.

### Determination of ATP power consumption with our ATP reporter and kinetic model

**Fig. 8:**
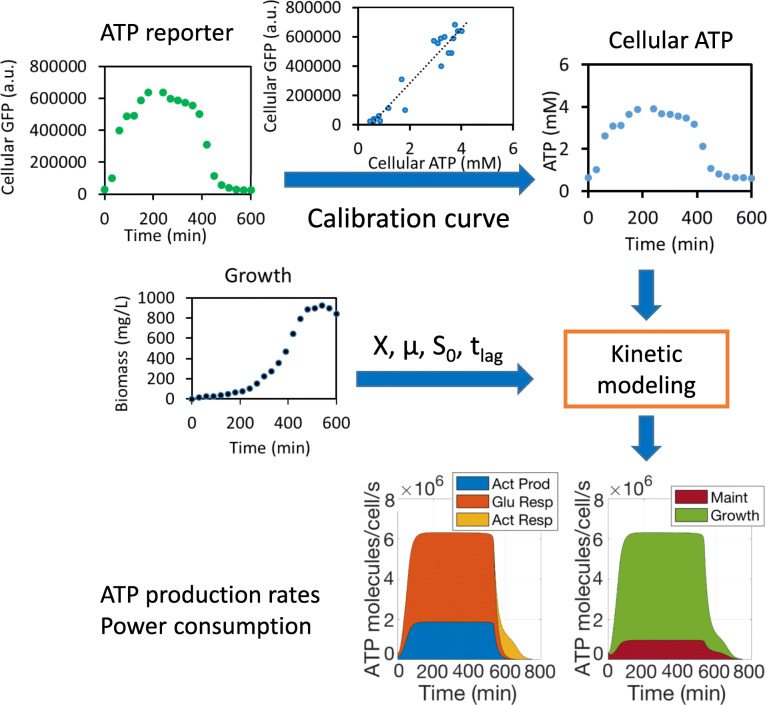
Using our ATP reporter and kinetic model to estimate cellular power consumption. GFP signals are measured from the reporter and converted to cellular ATP concentrations using a calibration curve pre-established under similar culture conditions. Bacterial growth is also measured, and parameters and initial conditions from this growth curve are used to determine the parameters of the kinetic model. ATP dynamics and cellular power consumption can then be reliably estimated without extra ATP measurements using the luciferase assay or other methods

As long as a calibration curve for ATP is pre-established under similar culture conditions, our ATP reporter and kinetic model enable us to determine cellular ATP levels and dynamic ATP consumption fluxes in living cells without the need for cell lysing, tedious ATP extraction, and costly luciferase assay procedures. We summarize how these determinations are made in Fig. 8. First, we measure GFP signals and convert them to cellular ATP concentrations using a pre-established calibration curve (e.g., Fig. 2) from similar experimental conditions. Then, our kinetic model is populated with parameters that are either known to be accurate for similar culture conditions from past experiments or derived by fitting the new experiment’s bacterial growth curve (growth rate, initial glucose concentration, initial cell population, and lag time). Finally, the GFP-reporter-derived ATP measurements and growth-curve parameters are then input to our kinetic model to calculate ATP consumption fluxes and thus cellular power consumption.

The accuracy of using our reporter to estimate ATP dynamic data were demonstrated by comparing those data with equivalent data obtained via a luciferase assay (Table 1). The closeness of those two datasets shows that the ATP dynamics and power consumption can be reliably predicted by the model and our ATP reporter without using a tedious and costly luciferase assay. As another example, using the methodology of Fig. 8 and a calibration curve for the BL21(DE3) strain (Additional file 5: Figure S5), we determined ATP dynamics and power consumption for this strain using just our reporter under the same condition (Additional file 11: Table S2). The model parameters for the BL21 strain were derived from those for the BW25113 strain (Fig. 6; Table 1) and needed only slight adjustments to account for strain-to-strain variations (Additional file 12: Table S3). In general, we found this strain grew faster than BW25113 strain, which contributed to higher ATP power consumption rates and faster ATP turnover during growth as estimated by our kinetic model using ATP input from our reporter.

## Discussion

In this study, we developed and validated an *rrnB* P1 promoter that can be used as an ATP reporter, and a kinetic model to measure ATP dynamics and ATP power consumption fluxes in *E. coli*. Our ATP reporter faithfully tracks cellular ATP dynamics in both the minimal and rich media across different growth phases and in different strains (Figs. 1, 2, 3, and 4). Although our results show that the HC-M ATP reporter works across different strains and conditions (Figs. 2 and 3, Additional file 5: Figure S5), we note that absolute accuracy requires that our ATP reporter be calibrated for different strains and conditions. In order to determine the dynamics and power consumption fluxes of ATP in a bacterial cell, we developed a dynamic model that accurately predicts bacterial growth, glucose and acetate metabolism, dissolved oxygen dynamics, and ATP dynamics (Fig. 5, the mathematical model in the “Methods” section, Additional file 6: Figure S6). Our results show that as long as the initial conditions (e.g., the amount of glucose and inoculum) are set and a maximum growth rate *μ*_max_ and lag time are determined from the experiment, ATP dynamics and fluxes can be accurately predicted directly from measurements and associated modeling without need for a luciferase assay or other methods for ATP measurement (Figs. 5, 6, 7, and 8). Even though experimental conditions can affect some constants and parameters, such constants can be pre-determined by additional experiments and our kinetic model is quite robust to various changes in experimental conditions (Fig. 6, Additional file 7: Figure S7).

High levels of the pheromone ppGpp can inhibit the activity of the *rrnB* P1 promoter and thus the GFP dynamics during starvation [18, 55]. As such, we did notice that relative GFP levels are a little lower than ATP levels during the stationary phase (Fig. 2, Additional file 5: Figure S5), when nutrients are low. However, by correlating GFP and ATP dynamics, we showed that such inhibition did not affect the operation of our ATP sensor significantly (Fig. 2). Our findings are in line with previous reports that *rrnB* P1 in strains without *spoT* and *relA* genes (no ppGpp) can also indicate cellular ATP levels [10, 56]. Furthermore, ATP and ppGpp in bacterial cells are anti-correlated such that they can cooperatively control the *rrnB* P1 promoter activity during different growth phases: at the start of the culture, cells have sufficient nutrients and thus ppGpp levels are very low while ATP accumulates rapidly. Consequently, ATP dominates the regulation of the activity of *rrnB* P1 during lag and exponential phases when nutrients are relatively abundant. In the stationary phase, as bacteria are experiencing nutrient shortage, cellular ATP levels drop but ppGpp accumulates to a higher level, which makes the promoter activity drop even further than may be expected by purely ATP-driven dynamics [56]. For these reasons, it is even possible that ppGpp might actually help ATP tracking in the nutrient-limited minimal medium: For strains short of ppGpp such as JM109(DE3), while we found a good correlation between GFP and ATP in rich medium (Additional file 5: Figure S5a,c), we did not find it in minimal medium, suggesting a potential role of ppGpp in this medium. However, given the similar GFP dynamics among four strains with likely different ppGpp backgrounds (Fig. 3), the effect of ppGpp on ATP tracking by our ATP sensor appears to be negligible in rich medium. For strains without ppGpp defect, our ATP reporter is found to be robust in both rich and minimal media, such as strains of BW25113 (Fig. 2) and BL21(DE3) (Additional file 5: Figure S5b,d).

Like almost all other transcriptional and translational biosensors, the expression of our ATP reporter could also be influenced by many factors other than ATP. Growth conditions, pH, and metabolites could all affect GFP dynamics. For example, low pH out of the physiological range might reduce the fluorescence of GFP [57]. Metabolites including ppGpp could affect the activity of *rrnB* P1 and the fluorescence of GFP. Despite the many factors that potentially impact GFP dynamics, the overall correlations between GFP and ATP of our biosensor are good in both the rich and minimal media over different growth phases and strains (Figs. 2 and 3). The calibration curves allow us to rapidly estimate ATP consumption rates and turnover times in bacteria without losing accuracy as compared to values obtained by luciferase assays and those in previous studies (Table 1). In addition, GFP dynamics could be influenced by plasmid copy number, strength of ribosome binding site (RBS) (Additional file 1: Figure S1), GFP degradation rate, energy source, and cellular nutritional status. In practice, many of these factors are tunable for optimization; for example, RBS strength can be altered by using the RBS calculator [58]; GFP degradation rate can also be engineered by using SsrA tags with different time constants [59]. Just as we have shown in this study, optimization may be needed to get good correlation between *rrnB* P1-GFP and ATP dynamics in strains or species considerably different from ours, but the methodology needed to obtain good correlation can likely benefit from our study and methods.

Notably, bacteria change their ATP pool very rapidly during growth, typically within 1 s. We found that each *E. coli* cell consumed ATP at the rate of around 6.4 million ATP/s during exponential growth under the experimental conditions tested and that consumption slowed approximately by 8–28-fold during lag and stationary phases, respectively (Fig. 7, Table 1). Assuming each cell has ~ 3.5 mM ATP during exponential growth (Fig. 6), the cellular ATP pool is turned over in ~ 0.3 s. Our measurements are consistent with previous reports that a growing *E. coli* cell turns over its entire ATP pool within 1 s depending on growth conditions [51]. Because of the high turnover rate, ATP production rate and consumption rate are balanced as predicted by our model (Fig. 7), which is important for maintaining ATP homeostasis in the cell [8, 51]. In terms of power consumption, it is amazing to note that each bacterial cell is extremely efficient in energy usage during growth. Our model predicts that an *E. coli* cell consumes only 0.57 pW during exponential growth, consistent with values reported previously [39, 41, 51]. Such power consumption is orders of magnitude more energy-efficient than today’s most advanced electronic devices in that cells can quickly adjust a protein/metabolite to a desired level in response to environmental disruptions, which for comparable speed and precision in electronic circuits performing similar signal processing would need at least a mW of power [39, 41].

Determining ATP dynamics is essential for studying molecular mechanisms of persistence, virulence, and gene regulation in bacteria under different conditions and stresses [7, 8, 10, 34, 35]. For example, ATP dynamics can provide valuable insights into bacterial metabolism and bacterial persistence to antibiotic drugs [10, 60–62]. Due to the multitude of variables affecting growth, using growth rate as a single indicator is insufficient to evaluate antibiotic efficacy towards pathogens especially when such pathogens are within hosts. Rather, it is the metabolic state of bacteria that largely influences their susceptibility to antibiotics [60–62]. Therefore, cellular ATP level as an indicator of bacterial metabolism can be and has been used to predict bacterial persistence or susceptibility to drugs [10, 60–62]. For example, above a certain threshold concentration, cellular ATP levels in the bacteria correlate negatively with bacterial survival rate from antibiotic treatment [11]. The combined use of our *rrnB* P1 ATP reporter and dynamic model facilitates the rapid determination of cellular ATP level and ATP power consumption fluxes in bacteria, which together give insight into bacterial metabolism and drug response. Moreover, ATP power consumption flux measurements may aid experiments in altering bacterial metabolism, which have been proposed as a novel strategy to enhance antibiotic treatments [60–62]. As we have shown here, ATP dynamic parameters such as the power consumption rate and turnover time might be better indicators of metabolic state than the absolute ATP concentration, which is homeostatically regulated by bacteria, and does not change as much. The easy combinatorial use of our ATP reporter and kinetic model may thus offer a useful tool for many fundamental microbiological studies as well as for synthetic biological applications.

## Conclusions

In this work, we designed a synthetic *rrnB* P1-GFP reporter and validated its use to measure ATP in *E. coli*. Regardless of many factors that might affect fluorescence-reporter dynamics that are common to almost all biosensors, good correlations were found between GFP and ATP under many experimentally tested conditions. We found that our ATP reporter can reliably monitor cellular ATP dynamics in response to nutrient availability. We developed a dynamic model that accurately predicted the dynamics of growth, glucose consumption, acetate production and consumption, oxygen consumption, and ATP production and consumption in living microbial cells. Using GFP signal as a proxy for ATP, our *rrnB* P1-GFP reporter and dynamic model together provide a fast and simple way to predict ATP power consumption fluxes in bacteria. We quantitatively demonstrated that, during exponential growth, bacteria turn over their ATP pool within a second, much faster than during lag and stationary phases and that the power consumption of bacteria during exponential growth was at least 8-fold greater than in other growth phases. We envisage that our ATP reporter and dynamic model may prove directly useful in studies of bacterial metabolism and shed insight into cellular power consumption-related effects that are known to be important in several diseases.

## Methods

### Strains, media, and growth conditions

The *E. coli* NEB 10-beta strain (New England BioLabs, Inc.) was used to construct all plasmids and for the quick screen of ATP reporters. We also tested the JM109 (DE3) strain that is deficient in ppGpp production due to the mutated *relA* gene, the BW25113 that is the parental strain of the Keio knockout collection and has wild-type genes for ppGpp production [63] and the BL21(DE3) strain also with wild-type ppGpp production genes.

The MOPS minimal medium (TEKnova Inc., cat#M2106) and MOPS EZ rich defined medium (TEKnova Inc., cat#M2105) were used for all ATP experiments and kinetic studies. Glucose was supplemented as the carbon/energy source at different concentrations noted by experiments. Unless otherwise noted, glucose in all our experiments (less than 15 mM for minimal medium or 10 mM for the rich medium) is the rate-limiting factor for bacterial growth. Minimal medium also received 0.2 mM of leucine to promote growth. Carbenicillin at 50 μg/ml and/or kanamycin at 50 μg/ml were supplemented before experiments. The final pH values of the cultures in all experiments were kept above pH 7.3 to minimize the potential pH effect on GFP fluorescence.

For ATP calibration and kinetic studies, a fresh single colony was inoculated in the minimal medium for 24 h or the rich medium for 16 h at 37 °C with shaking. The seed culture was then diluted 60–100 fold in 200 ml of the pre-warmed fresh medium in a 500-ml flask. The culture was incubated at 37 °C with shaking at 200 rpm. Samples were taken at different time points and immediately subjected to flow cytometry for GFP measurements and ATP extraction. When needed, a pre-calibrated Clark oxygen meter (Seven2Go DO meter, Mettler Toledo Inc., cat#30207959) was inserted into the culture and secured at the opening of the flask. This probe remained in the culture during the whole experiment and allowed for the continuous monitoring of dissolved oxygen. The supernatants of cultures were stored at − 20 °C until they were assayed for glucose and acetate as described elsewhere in this paper.

Experiments were also conducted in clear-bottom black 96-well plates (Corning Inc., cat#3603). Seed cultures were prepared as described above and diluted in either the fresh rich medium or minimal medium. Each well received 110 μl of diluted seed culture. The plate was then incubated in the SpectraMax Paradigm microplate reader (Molecular Devices, Inc.) at 37 °C with shaking. Bacterial growth (OD600) and GFP (ex 485/em 528) were recorded every 10 min. The cellular GFP signal was calculated as GFP/OD. Bacterial strains without any plasmids were also grown and the autofluorescence was measured and subtracted from the GFP signals.

### Construction of *rrnB* P1 ATP reporters and control

A fast-folding GFP (GFP-mut2) [25] was transcriptionally fused with an *rrnB* P1 promoter and an SsrA protease tag (LAA) for rapid degradation [26, 27]. The ribosome binding site (RBS) sequence was designed by the RBS calculator [58] to adjust the GFP expression rate. All reporters designed and used in this study are shown in Table 2. The high-fidelity Q5 DNA polymerase (NEB Inc.) was used to run all PCR reactions. The DNA fragments of the *rrnB* P1 promoter, RBS, GFP-mut2, LAA degradation tag, and backbone overlapping region were synthesized as one gBlock by IDT (Integrated DNA Technologies, Inc.) and assembled into either high-copy or low-copy plasmids using NEBuilder® HiFi DNA Assembly kit (NEB, Inc.). Two low-copy-plasmid reporters (LC-F and LC-G) with medium and high RBS strength were made; the backbone of pSC101 Ori with a kanamycin resistance gene was PCR amplified from the plasmid pRD123 [40], and the primer set used was HLCN-bk-F (5′- *TAACCCGGGGGATCCCATGGTA*-3′) and HLCN-bk-R (5′-*AGGTGGCACTTTTCGGGGAA-3′*). Two high-copy-plasmid reporters (HC-E and HC-M) with medium and low RBS strength were also made by fusing the same gBlock with the backbone from an HCN plasmid JF72 containing ampicillin resistance and the ColE1 Ori [64]. The high-copy plasmid backbone was PCR amplified using the same primer sets (HLCN-bk-F and HLCN-bk-R) as used for the low-copy plasmid. The assembled products were transformed into the NEB 10-beta strain (NEB, Inc., cat# C3019H) and selected for kanamycin- or ampicillin-resistant colonies on LB plates, for low-copy and high-copy plasmids, respectively. A strong constitutive promoter T7A1 [65, 66] was used to replace the *rrnB* P1 promoter from HC-E to make the plasmid HC-con as a control. One DNA fragment was PCR amplified from HC-E, using two primers, rrnL-R (5′-***AGTCAATACTCTTTTTGATAA****GACGTCAGGTGGCACTTTTCGGGGAA*-3′) and rrnL-F (5′-***TTATCAAAAAGAGTATTGACT***TAAAGTCTAACCTATAGGATACTTACAGCC*AGAATTCACCGATATCCGAACG*-3′). The primer set has overlapping sequences (bolded above) and a T7A1 promoter sequence. The DNA sequences of all three plasmids were confirmed by Sanger sequencing. The plasmids used in this work are shown in (Additional file 13: Figure S10) and the DNA sequences of promoters, RBS, GFP-mut2, and the degradation tag were shown in (Additional file 14).

**Table 2:** ATP reporter constructs designed and used in this study

Name	Plasmid copy number	RBS strength design
LC-F	Low copy	16,000, medium strength
LC-G	Low copy	50,000, high strength
HC-M	High copy	7000, low strength
HC-E	High copy	16,000, medium strength
HC-con	High copy	16,000, medium strength

The RBS strength is indicated as the translation initiation rate (arbitrary unit) designed by the RBS calculator and is arbitrarily defined as low, medium, and high strength in this study. All reporters were made with the *rrnB* P1 promoter except the control plasmid HC-con that used the strong constitutive promoter T7A1

### Flow cytometry

The GFP signal was measured by a CytoFLEX S Flow Cytometer (Beckman Coulter, Inc.) or a microplate reader (Molecular Devices, Inc.). For flow cytometry, bacterial samples were diluted into phosphate-buffered saline (PBS) and immediately subjected to flow cytometry. Cells were gated by forward scatter (FSC) and side scatter (SSC), and at least 20,000 events were collected for each sample. Green fluorescence was collected using the FITC channel. The mean fluorescence intensity (MFI) of each cell was calculated and used to make GFP/cell dynamic curves. The autofluorescence of bacterial cells without any plasmids was also collected and subtracted for each sample. All flow cytometry data were analyzed by FlowJo v10 (TreeStar Inc., Ashland, OR). To estimate the cell count density (cells/L), the AccuCheck counting beads (Molecular Probes Inc., cat# PCB100) with a known density (beads/L) were run with bacterial samples and the absolute cell count density was calculated. Biomass measurements are products of cell count density and the cell dry mass (405 fg/cell) for bacteria grown in the minimal medium [67].

### ATP extraction and luciferase assay

Bacteria samples taken at different time points were immediately subjected to ATP extraction by ice-cold perchloric acid that simultaneously lyses cells, stops metabolism, and stabilizes ATP [68, 69]. The ATP extracts were stored at − 20 °C for no more than 5 days before they were analyzed. Before the luciferase assay, the samples were neutralized by an ice-cold buffer containing 0.72 M KOH and 0.16 M KHCO_3_, and the supernatants were diluted three-fold in 100 mM Tris buffer (pH 7.8). ATP samples were quantified by luciferase assay using an ATP Bioluminescence Assay Kit (Roche Inc., cat# 11699695001), with blank media subjected the same preparation above as the control. Cellular ATP concentrations were calculated from cell count density and cell volume using the formula: ${\mathrm{ATP}}_{\mathrm{c}\mathrm{ell}}=\frac{{\mathrm{ATP}}_{\mathrm{pop}}}{{\mathrm{N}}_{\mathrm{c}}\ast {V}_{\mathrm{c}\mathrm{ell}}}$, where *N*_*c*_ is the cell count density determined by flow cytometry and *V*_cell_ is the cell volume (assuming 1 fL/cell and 1.4 fL/cell for bacteria grown in minimal and rich media, respectively) [70].

### Dissolved oxygen measurement

Dissolved oxygen (DO) was measured by the Seven2Go DO meter as mentioned above. To determine the volumetric mass transfer coefficient (k_L_a), we used fresh blank medium under the same conditions used for kinetic studies. The oxygen transfer rate (OTR) from the gas phase to the liquid phase follows a simple equation [71] and can be described by $\frac{dDO}{dt}={k}_La\ast \left(D{O}^{\#}- DO\right)$, where DO is the dissolved oxygen concentration in the medium and DO^#^ is the saturated DO. To create a low-oxygen medium, around 1.5 ml of 100 mM sodium sulfite solution was added to 200 ml of the blank medium at room temperature, followed by sitting in the incubator at 37 °C for at least 1 h to reach temperature and DO equilibrium. This ensured that most oxygen was removed from the medium while no excess sodium sulfite remained that could affect the k_L_a measurement. Dissolved oxygen data was collected and used to determine k_L_a by running a simulation in the kinetic circuit model described below without oxygen consumption.

### Glucose and acetate assays

Glucose levels in culture supernatants at different time points were determined by a 3,4-dinitrosalicylic acid (DNS) method in 96-well microplates [72, 73]. Acetate in the supernatants was assayed by an Acetate Colorimetric Assay Kit (BioVision Inc., cat# K658100) according to instructions from the manufacturer. Blank minimal and rich media were included in all assays as blank controls.

### Kinetic model development

Bacteria were grown in the minimal medium with glucose as the sole carbon source and hence the rate-limiting substrate. The kinetic model was developed based on the schematic workflow (Fig. 5) that depicts carbon fluxes and ATP fluxes for bacterial growth. Bacterial growth kinetics are described by the classic Monod equation, which results in the following relationships:

1 $$ \frac{dX}{dt}=\mu X=\Big\{{\displaystyle \begin{array}{c}{\mu}_sX={\mu}_{\max, s}\frac{S}{K_S+S}X,S>0\\ {}{\mu}_AX={\mu}_{\max, A}\frac{A}{K_A+A}X,S=0\end{array}}\operatorname{} $$

where *S* is glucose concentration (M), *A* is acetate concentration (M), *μ*_*S*_ and *μ*_*A*_ are the specific growth rates (1/s) for growth on glucose and acetate, respectively, X is the biomass concentration (g/L), and *t* is the time (s). X is determined as the product of cell mass [67] and cell counts/L. The specific growth rates are determined by Monod equations where *μ*_max,*S*_ and *μ*_max,*A*_ are the experimentally measured maximal specific growth rates on glucose and acetate respectively, and K_S_ and K_A_ are the Monod saturation constants for glucose and acetate, respectively. Bacterial growth starts after a brief lag time that is determined by specific experiments.

Acetate is produced and secreted when *E. coli* cells aerobically grow on excessive glucose. This behavior is called overflow metabolism or energy spilling, a well-known phenomenon that is caused by surplus glucose uptake in fast-growing bacteria in the presence of oxygen [44, 45, 74, 75]. The secreted acetate is then utilized by the bacteria after glucose is nearly totally consumed, undergoing a process known as the “acetate switch” [76]. Therefore, in our model bacterial growth (*μ*_*A*_) on acetate is turned on only after glucose is exhausted, i.e., when *S* = 0. In practice in our circuit simulation, growth on acetate is turned on when *S* is nearly depleted to zero (*S* < *S*_tran_, with *S*_tran_ = 0.1 mM) (Additional file 8: Table S1), which is biologically relevant, and which ensures a relatively smooth transition between glucose metabolism and acetate utilization, seen by others [76] and in fitting our measured biological data. Since this period is rather short and does not have any significant effects on our overall model dynamics or results, it has been omitted from the equations for simplicity and clarity. Acetate was reported to inhibit bacterial growth at high concentrations [45, 77]; but, under our experimental conditions, its concentration was so low that this inhibitory effect is negligible and ignored in our model.

Oxygen is supplied through agitation and shaking and is consumed by aerobic respiration of glucose, conversion of glucose to acetate, and aerobic respiration of acetate during growth. The oxygen supply parameter (*k*_*L*_*a*) was pre-determined by experiments described above. Our model assumes that oxygen consumed for aerobic respiration and acetate production is proportional to bacterial growth. There are two stages of growth using glucose and acetate, respectively. When glucose is available, the bacteria consumes oxygen for aerobic respiration and for acetate production using glucose (Fig. 5a); when glucose is exhausted, the bacteria consume oxygen for aerobic respiration using secreted acetate. The stoichiometry of acetate production follows the biochemical pathways of central carbon metabolism and acetate metabolism (Fig. 5a) and the overall biochemical reaction is shown below:

$$ {\mathrm{C}}_6{\mathrm{H}}_{12}{\mathrm{O}}_6\ \left(\mathrm{glucose}\right)+2\ {\mathrm{O}}_2\to 2\ {\mathrm{C}\mathrm{H}}_3\mathrm{COOH}\ \left(\mathrm{acetate}\right)+2\ {\mathrm{C}\mathrm{O}}_2+2\ {\mathrm{H}}_2\mathrm{O}+14\ \mathrm{ATP} $$

Therefore, the dynamics of dissolved oxygen are described by the following equations:

2 $$ \frac{d\mathrm{DO}}{dt}={k}_La\left({\mathrm{DO}}^{\#}-\mathrm{DO}\right)-\mathrm{OCR} $$

3 $$ \mathrm{OCR}=\Big\{{\displaystyle \begin{array}{c}{k}_{\mathrm{occ},S}\ {\mu}_SX+{k}_{A,\mathrm{pro}}\ {\mu}_SX,\kern0.28em S>0\\ {}{k}_{\mathrm{occ},A}\ {\mu}_AX,S=0\end{array}}\operatorname{} $$

where DO is the dissolved oxygen in the medium in molar concentration (M) and DO^#^ is the saturated dissolved oxygen concentration (M), *k*_*L*_*a* is the experimentally determined volumetric oxygen transfer coefficient (1/s), and OCR is oxygen consumption rate (M/s). OCR has two main phases dependent on glucose availability in Eq. (3) where *k*_*occ*,*S*_ and *k*_*occ*,*A*_ are oxygen consumption coefficients (mol/g cells) for growth on glucose and acetate, respectively, and *k*_*A*,*pro*_ is the oxygen consumption rate coefficient (mol/g cells) for acetate production. Since DO can be measured continuously and the oxygen supply rate is pre-determined by experiments under the same conditions, oxygen consumption rates for glucose and acetate metabolism can be determined.

Glucose is used for biomass synthesis, aerobic respiration, and acetate production and thus has three fluxes to describe its dynamics (Fig. 5). The glucose consumption rate for biomass synthesis can be determined from the mass balance of carbon between glucose and biomass:

4 $$ \frac{d{S}_{bs}}{dt}=-\frac{\alpha }{72}{\mu}_SX $$

where *S*_*bs*_ (M) is the glucose that goes to biosynthetic flux and α is the proportion of carbon in biomass. Based on the stoichiometry from the elemental composition of *E. coli* cells (CH_1.61_N_0.27_O_0.41_S_0.006_P_0.019_) [78], we calculated α = 0.485. Because each carbon has a molar mass of 12 g and glucose has six carbons, biosynthesis of each gram of biomass consumes $\frac{\alpha }{72}$ moles of glucose.

Our model assumes that acetate production rate is proportional to growth rate [79–81], so the acetate production rate is defined as *k*_*A*,*pro*_*μ*_*S*_*X*, where *k*_*A*,*pro*_ is the acetate production rate constant (mole/g cells). According to the stoichiometric relationships in the acetate production pathway (Fig. 5a), each mole of acetate produced consumes 1/2 mol of glucose. For aerobic respiration of glucose, one mole of oxygen consumes 1/6 mol of glucose. Together with Eq. 4, the total glucose consumption rate is described as:

5 $$ \frac{dS}{dt}=\Big\{{\displaystyle \begin{array}{c}-\frac{\alpha }{72}{\mu}_SX-\frac{1}{6}{k}_{\mathrm{occ},S}\ {\mu}_SX-\frac{1}{2}{k}_{A,\mathrm{pro}}\ {\mu}_SX,\kern0.56em S>0\\ {}0,\kern0.56em S=0\end{array}}\operatorname{} $$

The dynamics of acetate include its production from glucose, and its consumption for biomass synthesis and aerobic respiration (Fig. 5a). When glucose is available, acetate production is defined as *k*_*A*,pro_*μ*_*S*_*X* in Eq. 5. From the mass balance of carbon between acetate and biomass, synthesis of each gram of biomass consumes $\frac{\alpha }{24}$ moles of acetate. One mole of oxygen consumes 1/2 mol of acetate for aerobic respiration. Bringing together the acetate production, biomass synthesis, and aerobic respiration, we have the equation below:

6 $$ \frac{dA}{dt}=\Big\{{\displaystyle \begin{array}{c}{k}_{A,\mathrm{pro}}\ {\mu}_SX,S>0\\ {}-\frac{\alpha }{24}{\mu}_AX-{k}_{\mathrm{occ},A}\ {\mu}_AX,S=0\end{array}}\operatorname{} $$

where *α* is the same as defined in Eq. (4), *k*_*A*,pro_ is the acetate production rate constant and *k*_occ,*A*_ is the oxygen consumption coefficient (mole/g cells) for acetate, as described in Eq. (3).

The above Eqs. 1–6 describe all mass fluxes including biomass, oxygen, glucose, and acetate (Fig. 5a). Those parameters can be measured in experiments and thus the fluxes can be resolved by solving the above ordinary differential equations using a software like MATLAB. However, we took advantage of electrical circuits, which can exactly match, simulate, and visualize differential equations, as evident in our previous publications [37–42, 82]. The circuits can be easily designed and simulated using the classic electrical engineering software, Cadence Virtuoso (Cadence Design Systems, Inc.). The circuits that match and visualize all equations in this work are shown in the supplementary materials (Additional file 6: Figure S6).

### ATP dynamics model

After validating the above model by fitting it to experimental data, we then developed equations for ATP dynamics from the model above. To determine ATP dynamics, ATP concentrations and fluxes on both the cellular and population levels must be considered and unified. The conversion between the two levels is:

7a $$ {\mathrm{ATP}}_{\mathrm{pop}}=\frac{X\ }{\rho_{\mathrm{cell}}}\kern0.50em {\mathrm{ATP}}_{\mathrm{cell}} $$

7b $$ {I}_{\mathrm{ATP},\mathrm{pop}}=\frac{X\ }{\rho_{\mathrm{cell}}}\kern0.50em {I}_{\mathrm{ATP},\mathrm{cell}} $$

where *ρ*_cell_ is the cell density (g/L) that is estimated from division of cell mass (*m*_cell_) [67] by cell volume (*V*_cell_) [70], ATP_pop_ and ATP_cell_ are the population ATP concentration (moles/L culture) and the cellular ATP concentration (moles/L cell volume), respectively, and *I*_ATP,pop_ and *I*_ATP,cell_ are a population ATP flux (moles/s/L culture) and a cellular ATP flux (moles/s/L cell volume).

The overall ATP accumulation in the cell is defined by the equation below:

8 $$ \frac{{\mathrm{dATP}}_{\mathrm{cell}}}{dt}=\frac{\rho_{\mathrm{cell}}}{X}\left({I}_{\mathrm{ATP},\mathrm{pro},\mathrm{pop}}\right)-{I}_{\mathrm{ATP},\mathrm{con},\mathrm{cell}} $$

where *I*_ATP,pro,pop_ is the ATP production rate at population level, and *I*_ATP,con,cell_ is the ATP consumption rate at the cellular level. *I*_ATP,pro,pop_ is the sum of all ATP production rates (moles/s/L culture) from fluxes of glucose aerobic respiration (*I*_resp_), acetate production (*I*_A,pro_), and acetate respiration (*I*_*A*,*resp*_) when glucose is depleted (Fig. 5a), and thus is described by the following:

9 $$ {I}_{\mathrm{ATP},\mathrm{pro},\mathrm{pop}}=\Big\{{\displaystyle \begin{array}{c}{I}_{\mathrm{resp}}+{I}_{A,\mathrm{pro}},S>0\\ {}{I}_{A,\mathrm{resp}},S=0\end{array}}\operatorname{} $$

These fluxes for glucose and acetate are defined in Eqs. 5 and 6. Now, we need to convert them to ATP production rates by using stoichiometric ATP yields. The ATP production rate from glucose respiration is the product of a stoichiometric constant and the rate of glucose consumption for aerobic respiration:

10 $$ {I}_{\mathrm{resp}}=\frac{16}{3}{k}_{\mathrm{occ},S}\ {\mu}_SX $$

where $\frac{16}{3}$ is the stoichiometric constant, representing the 32 ATP molecules yielded from 6 oxygen molecules consumed when one glucose molecule is used [83] with a P/O ratio of 2.67. Due to the imperfect efficiency of ATP generation system such as proton leakage [83, 84], we used the conversion numbers for oxidative phosphorylation: NADH = 2.5 ATP and FADH = 1.5 ATP. The glucose consumption rate, *k*_occ,*S*_*μ*_*S*_*X*, for respiration is defined above in Eqs. 5 and 6.

During aerobic acetate production, consumption of one glucose molecule generates 2 acetate molecules and 14 ATP and consumes 2 di-oxygen molecules (Fig. 5a). This is because glycolysis yields 2 ATP, 2 NADH, and 2 pyruvate which are then converted to acetyl-CoA and produce two more NADH. Two acetyl-CoA then produce 2 ATP. This pathway yields 4 ATP from substrate level phosphorylation [45] and 4 NADH, equivalent to 10 more ATP from oxidative phosphorylation. Therefore, for every 2 acetate produced, we have 14 ATP generated and 1 glucose consumed:

11 $$ {I}_{A,\mathrm{pro}}=7{k}_{A,\mathrm{pro}}\ {\mu}_SX $$

The last flux of ATP production is from aerobic acetate respiration when glucose has been depleted and bacteria are growing on acetate. Acetate consumption rate is proportional to bacterial growth. Acetate is mainly utilized through the action of phosphate acetyl-transferase (Pta) and acetate kinase (AckA) in the Pta-AckA pathway or through the acetyl-CoA synthase (ACS) pathway to produce acetyl-CoA, consuming 1 ATP [85]. The acetyl-CoA then enters the tricarboxylic acid (TCA) cycle, yielding 3 NADH, 1 FADH and 1 GTP, together equivalent to 10 ATP. Therefore, the net ATP yield from acetate respiration is 9 ATP per acetate consumed, requiring 2 oxygen molecules. Based on the oxygen consumption rate for acetate respiration, *k*_occ,*A*_*μ*_*A*_*X*, we have the corresponding ATP production flux as below:

12 $$ {I}_{A,\mathrm{resp}}=\frac{9}{2}{k}_{\mathrm{occ},A}\ {\mu}_AX $$

Therefore, the total ATP production rate defined in Eq. 9 is the sum of the Eqs. 10–12. Together, we have:

13 $$ {I}_{\mathrm{ATP},\mathrm{pro},\mathrm{pop}}=\Big\{{\displaystyle \begin{array}{c}\frac{16}{3}{k}_{\mathrm{occ},S}\ {\mu}_SX+7{k}_{A,\mathrm{pro}}\ {\mu}_SX,S>0\\ {}\frac{9}{2}{k}_{\mathrm{occ},A}\ {\mu}_AX,S=0\end{array}}\operatorname{} $$

Once this population flux is converted to a cellular flux, a delay time constant (*τ*_delay_) is applied to each of the three terms in order to achieve a smooth transition between the exponential phase and the stationary phase (Additional file 8: Table S1). Such a time constant accounts for the time for gene turn-on/turn-off and associated protein-enzyme changes that may occur during a growth phase transition. These changes occur smoothly and not abruptly in real cells as well as in our measured biological data. A global single parameter value of 20 min for such first-order-time-constant dynamics (corresponding to typical cellular dynamics for protein rise and fall times in cells) was found to fit our data well.

We next resolved the ATP consumption rate, *I*_ATP,con,cell_. First, we made a model for cellular ATP consumption rate. The total cellular energy budget includes maintenance energy consumption and growth energy consumption [48–50]. The maintenance ATP consumption is often assumed to be a constant for easy implementation [86], but this is far from what may occur in real biological processes [49]. For simplicity, we used a maintenance energy consumption that is just proportional to cellular ATP levels. We found that it gave us the best fits and most accurate results for ATP dynamics, as also being verified by sensitivity analyses in the Results section. The assumption of first-order maintenance ATP consumption enables more robust ATP homeostasis such that ATP is used more when it is plentiful and is conserved when it is scarce. Such ATP homeostasis for cellular maintenance [48, 87] is especially important in the stationary phase when cells are not active and ATP is quite scarce. It is worth noting that, under such conditions, higher levels of ATP will cause more thermodynamic reactions in cells to be irreversible or to proceed more quickly, thus increasing consumption, naturally. Overall, the cellular energy consumption rate (mol ATP/s/L cells) is then described by:

14 $$ {I}_{\mathrm{ATP},\mathrm{con},\mathrm{cell}}=m\ {\mathrm{ATP}}_{\mathrm{cell}}+g\ {\mu}_{sm} $$

where *m* is the maintenance energy consumption rate coefficient (1/s), *g* is the growth-associated energy consumption coefficient (mol/L cell volume), and *μ*_*sm*_ is a version of *μ* = *μ*_*S*_ + *μ*_*A*_ that has both growth rate terms smoothed by time constants to allow for a steady transition from ATP consumption during growth using glucose to ATP consumption during growth using acetate. *m* and *g* are fitted to measured biological data.

Now, we can plug Eqs. 13 and 14 into Eq. 8 and simplify to obtain a model for cellular ATP dynamics:

15 $$ \frac{{d\mathrm{ATP}}_{\mathrm{cell}}}{dt}=\Big\{{\displaystyle \begin{array}{c}{\rho}_{\mathrm{cell}}\left(\frac{16}{3}{k}_{\mathrm{occ},S}\ {\mu}_S+7{k}_{A,\mathrm{pro}}\ {\mu}_S\right)-\left(m{\mathrm{ATP}}_{\mathrm{cell}}+g{\mu}_{sm}\right),S>0\\ {}{\rho}_{\mathrm{cell}}\left(\frac{9}{2}{k}_{\mathrm{occ},A}{\mu}_A\right)-\left(m{\mathrm{ATP}}_{\mathrm{cell}}+g{\mu}_{sm}\right),S=0\end{array}}\operatorname{} $$

Also, note that the cellular ATP consumption rate is resolved in Eq. 14. The power consumption per cell (ATPs/cell/s) can thus be calculated using the kinetic model. All parameters used in the model to create Fig. 6 are shown in Additional file 8: Table S1.

## Supplementary Information

**Additional file 1: Figure S1.** Quick screen for different ATP reporter constructs. (**a**) Normalized cellular GFP dynamics (%GFP/OD) of different ATP reporter constructs in rich medium. (**b**) Growth of the *E. coli* 10-beta strain carrying different reporter plasmids in rich medium. Bacteria were grown in EZ rich medium with 5 mM glucose in black 96-well plates with shaking. GFP (ex485/em528) and OD600 were measured with a microplate reader (Molecular Devices, Inc.) in real time. The cellular GFP signals, GFP/OD, were normalized by their own peak values (100%). Each data point is the mean value of at least three independent experiments with standard deviation less than 15% of its mean. All reporter constructs except HC-con incorporated the ATP-dependent *rrnB P1* promoter; the HC-con version was made with a sequence identical to HC-E except that a T7A1 promoter replaced the *rrnB* P1 promoter thus enabling it to serve as a control.

**Additional file 2: Figure S2.** GFP-ATP correlation analysis of the HC-E reporter in bacteria during growth. (**a**) GFP and ATP dynamics over the growth phases. NEB10beta strain with the HC-E reporter was grown in rich medium. ATP was measured by luciferase assay and cellular fluorescence was measured by flow cytometry. Data points are mean values of three independent replicates with one standard deviation (SD). The SD for GFP signal were relatively small (< 15%) and are thus not shown in the figure. (**b**) Linear correlation between GFP and ATP.

**Additional file 3: Figure S3.** Flow cytometry analysis of bacterial population with HC-M reporter growing in the EZ rich medium. Density plot and histogram plot of GFP populations at the lag phase (**a,b**), exponential phase (**c,d**), transition between exponential and stationary phases (**e,f**), and stationary phase (**g,h**). BW25113 strain with HC-M ATP reporter was analyzed. Cellular GFP was measured by FITC-A channel.

**Additional file 4: Figure S4.** Flow cytometry analysis of bacterial population with HC-M reporter growing in the minimal medium. Density plot and histogram plot of GFP populations in the lag phase (**a,b**), exponential phase (**c,d**), transition between exponential and stationary phases (**e,f**), and stationary phase (**g,h**). The BW25113 strain with HC-M ATP reporter was analyzed. Cellular GFP was measured by FITC-A channel.

**Additional file 5: Figure S5.** GFP-ATP correlation analysis of the HC-M reporter in two other strains. (**a,b**) GFP and ATP dynamics over the growth phases in the JM109DE3 strain in the rich medium (**a**) and in BL21DE3 strain in the minimal medium (**b**). ATP was measured by luciferase assay and cellular fluorescence was measured by flow cytometry. Data points are mean values of three independent replicates with one standard deviation (SD). The SD for the GFP signal was relatively small (< 15%) and is thus not shown in the figure. (**c,d**) Linear correlations between GFP and ATP in the JM109DE3 strain in the rich medium (**c**) and in BL21DE3 strain in the minimal medium (**d**).

**Additional file 6: Figure S6.** Electrical circuit model that visualizes and describes all differential equations in this study. The whole circuit includes bacterial growth (X), oxygen supply and consumption (DO), glucose dynamics (S), acetate production and consumption (A), and ATP production and consumption (ATP). ATP_pop_ is the amount of ATP in 1 L of culture while ATP_cell_ is the ATP concentration within the cell. The concentration of a substance is represented by the corresponding voltage. The consumption or production rate (flux) of a substance is represented by the corresponding current. A wire labeled with the same name as another wire means they are the same voltage (concentration) in the circuit. Initial concentrations are indicated by i.c. This circuit model also allows for the easy setting of timing mechanisms such as lag time, the level of glucose at which growth on acetate begins, and time constants to account for the smooth, non-instantaneous nature of switching between metabolic states. The capacitors (C), representing volume, are always normalized to 1 F to match the differential equations used to describe the time dynamics of concentrations. More detailed explanation is in the reference [43].

**Additional file 7: Figure S7.** Sensitivity analysis of the kinetic model by varying initial glucose and growth rate. Dynamics of bacterial growth (**a**), population ATP (**b**), and cellular ATP (**c**) at varying initial glucose concentration. Dynamics of bacterial growth (**d**), glucose consumption (**e**), and cellular ATP (**f**) at varying growth rates. The analysis was performed by varying the initial glucose concentration or growth rate while keeping other parameters identical to those obtained from experiments under the same conditions as those in Fig. 6. Increasing the specific growth rate from 0.4 to 0.7 (1/h) needs the slight adjustment of *g* from 54.5 to 60.7 (M) for the growth rate sweep.

**Additional file 8: Table S1.** Model parameters for *E. coli* strain BW25113 grown in minimal medium.The values of K_S_, t_lag_ and k_L_a are supported by the references [88–90], respectively.

**Additional file 9: Figure S8.** Comparison of cellular oxygen consumption rate and ATP production rate. Both oxygen flux and ATP production flux were determined from our kinetic model using the experimental data used in Fig. 6.

**Additional file 10: Figure S9.** Model response to varying initial cellular ATP concentrations. All model parameters except the initial cellular ATP fluxes from acetate production and aerobic respiration of glucose were held constant while initial cellular ATP concentration was varied. The initial ATP fluxes change linearly with the initial ATP concentration because we assumed that a cell with a higher initial ATP concentration is in a healthier metabolic state and will initially be producing ATP at a higher rate.

**Additional file 11: Table S2.** Calculated ATP values in *E. coli* BL21(DE3) grown in the minimal medium. a: Power consumption was estimated from 54 kJ/mole ATP [53, 54]. Note: all values are estimated from one biological experiment with three samples measured at each time point. In this experiment, ATP concentration was measured by the HC-M reporter and ATP consumption rates were calculated by the kinetic model.

**Additional file 12: Table S3.** Model parameters of the model for *E. coli* strain BL21(DE3) grown in minimal medium. These parameters are used in the simulation that calculates the dynamic ATP values in Additional file 11: Table S2. These parameters are same to those used for the BW25113 strain with only slight changes for a few parameters to account for strain-to-strain variations.

**Additional file 13: Figure S10.** Plasmids constructed and tested in this work. (**a**) High-copy-plasmid, low RBS reporter HC-M. (**b**) High-copy-plasmid, medium RBS reporter HC-E. (**c**) High-copy-plasmid control reporter HC-con with T7A1 constitutive promoter. (**d**) Low-copy-plasmid, medium RBS reporter LC-F. (**e**) Low-copy-plasmid, high RBS reporter LC-G. High-copy plasmids have a ColE1 origin of replication while the low-copy plasmids have a PSC101 origin of replication.

**Additional file 14.** The inserted DNA sequences of the plasmids used in this study.

**Additional file 15.** Experimental data for Figs. 1, 2, 3, 4, 6, and Figures S1, and S5.

**Additional file 16.** Experimental data and parameters used for Table 1, Table S2, and model analyses.

**Additional file 17.** Cadence files for the kinetic model circuit. The .zip file contains the Cadence library of components (schematics and symbols) and cellview simulation states needed to perform the kinetic model simulations presented in this paper.

## Data Availability

All supporting data generated or analyzed during this study are included in this published article and its supplementary files (Additional files 15, 16). The DNA sequences of the plasmids used in this study are in Additional file 14. The Cadence simulation files for the kinetic model circuit are in the Additional file 17. The authors will make the plasmids available through public repositories so that any requests for them can be fulfilled.
