# Supplementary figures and images for: Measuring and modeling energy and power consumption in living microbial cells with a synthetic ATP reporter
Source: BMC Biol. 2021 May 17;19:101. doi: 10.1186/s12915-021-01023-2 (PMC8130387; doi:10.1186/s12915-021-01023-2)

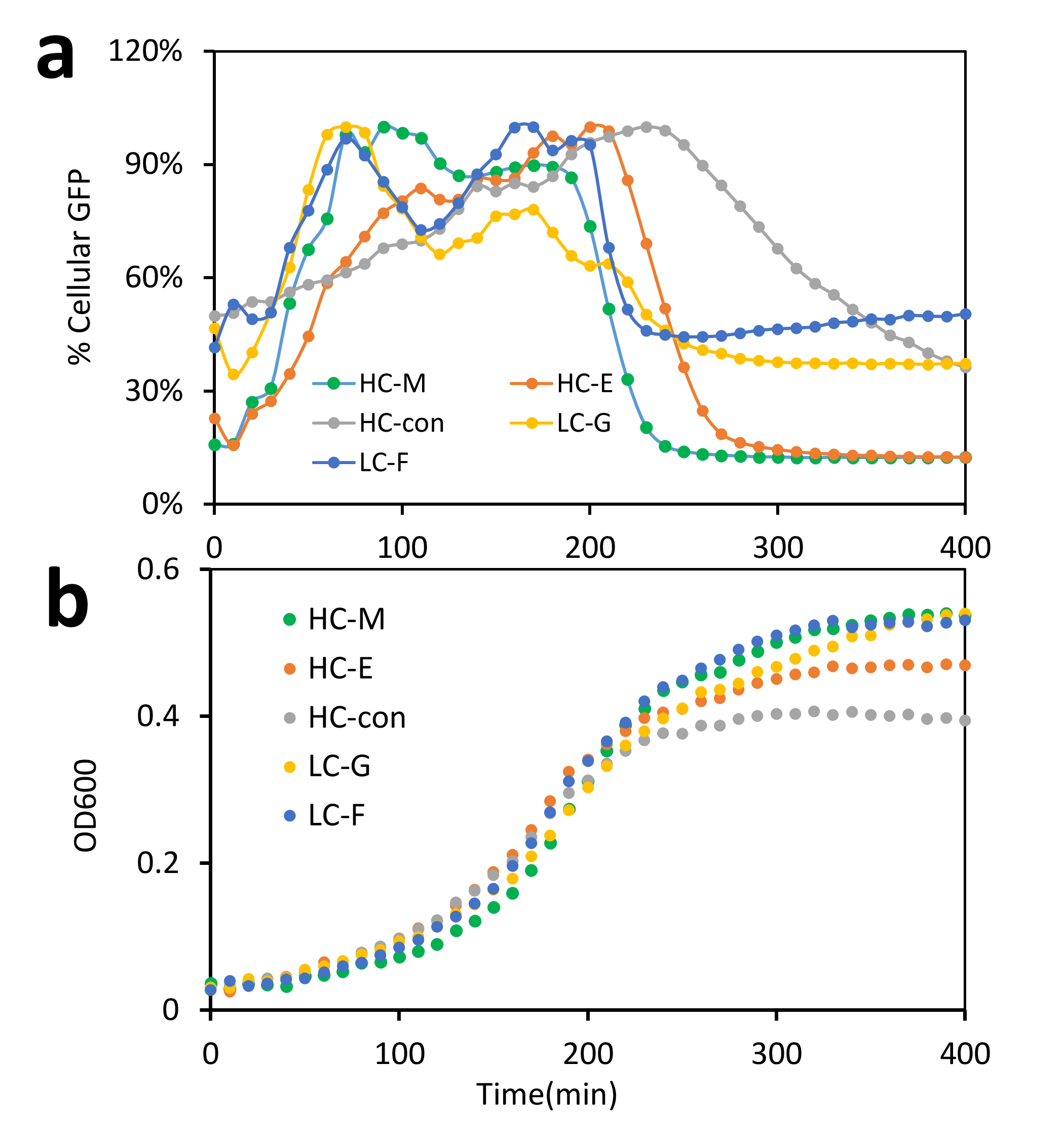

Supplement: Supplementary file 1 — Additional file 1: Figure S1. Quick screen for different ATP reporter constructs. (a) Normalized cellular GFP dynamics (%GFP/OD) of different ATP reporter constructs in rich medium. (b) Growth of the E. coli 10-beta strain carrying different reporter plasmids in rich medium. Bacteria were grown in EZ rich medium with 5 mM glucose in black 96-well plates with shaking. GFP (ex485/em528) and OD600 were measured with a microplate reader (Molecular Devices, Inc.) in real time. The cellular GFP signals, GFP/OD, were normalized by their own peak values (100%). Each data point is the mean value of at least three independent experiments with standard deviation less than 15% of its mean. All reporter constructs except HC-con incorporated the ATP-dependent rrnB P1 promoter; the HC-con version was made with a sequence identical to HC-E except that a T7A1 promoter replaced the rrnB P1 promoter thus enabling it to serve as a control. [file 12915_2021_1023_MOESM1_ESM.tiff]

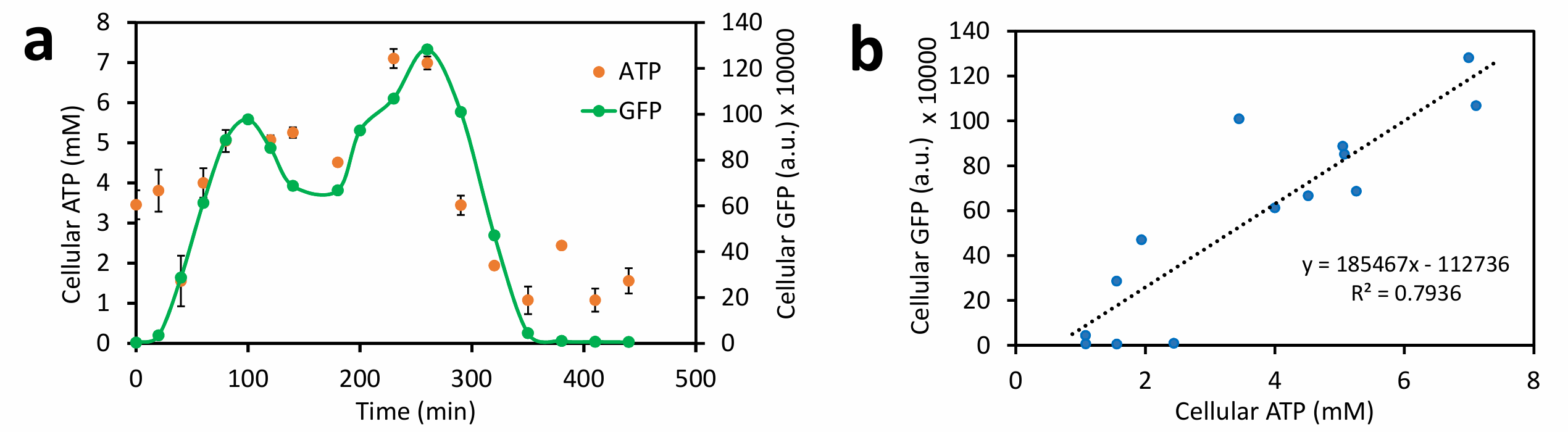

Supplement: Supplementary file 2 — Additional file 2: Figure S2. GFP-ATP correlation analysis of the HC-E reporter in bacteria during growth. (a) GFP and ATP dynamics over the growth phases. NEB10beta strain with the HC-E reporter was grown in rich medium. ATP was measured by luciferase assay and cellular fluorescence was measured by flow cytometry. Data points are mean values of three independent replicates with one standard deviation (SD). The SD for GFP signal were relatively small (< 15%) and are thus not shown in the figure. (b) Linear correlation between GFP and ATP. [file 12915_2021_1023_MOESM2_ESM.tif]

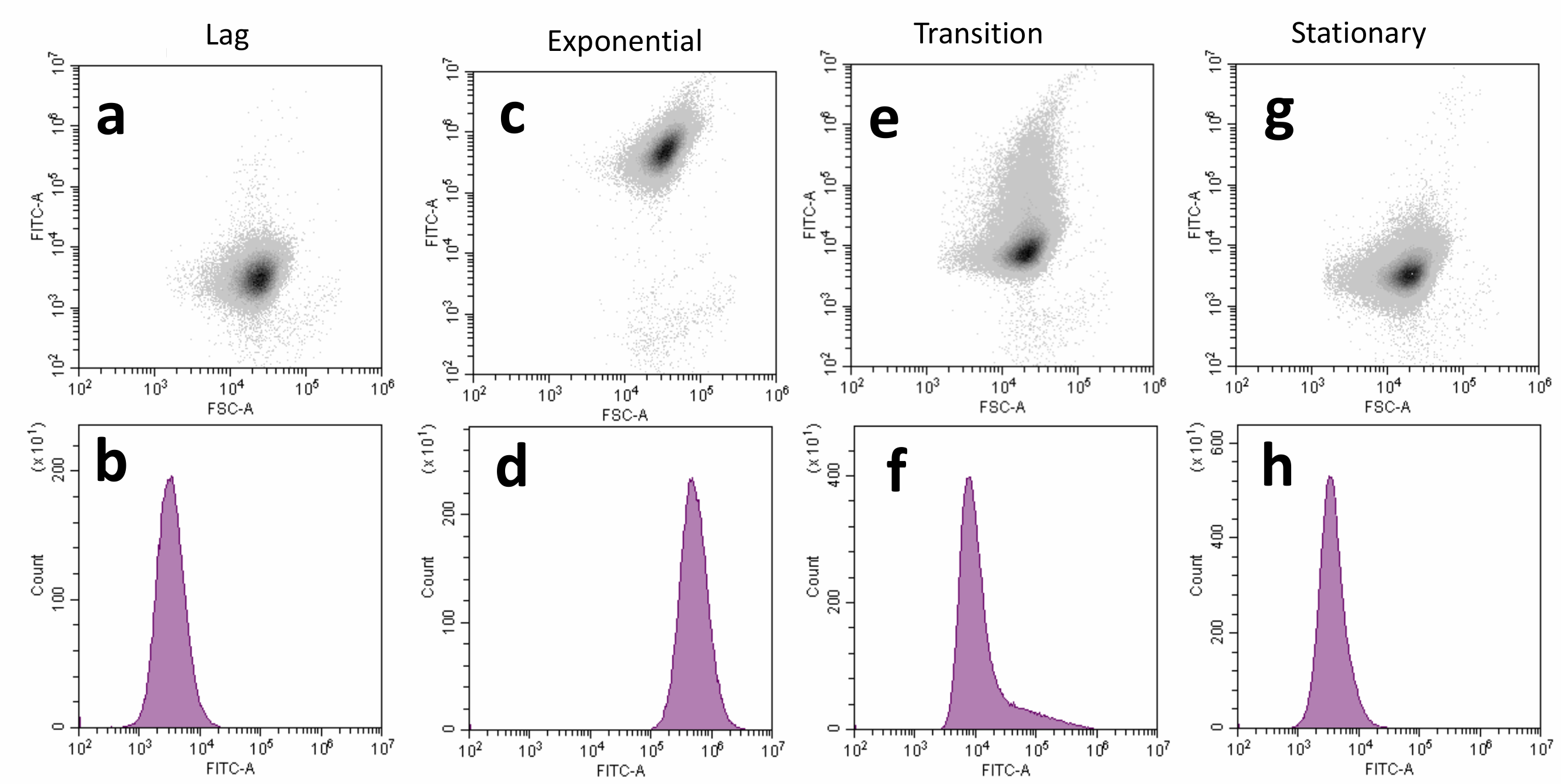

Supplement: Supplementary file 3 — Additional file 3: Figure S3. Flow cytometry analysis of bacterial population with HC-M reporter growing in the EZ rich medium. Density plot and histogram plot of GFP populations at the lag phase (a,b), exponential phase (c,d), transition between exponential and stationary phases (e,f), and stationary phase (g,h). BW25113 strain with HC-M ATP reporter was analyzed. Cellular GFP was measured by FITC-A channel. [file 12915_2021_1023_MOESM3_ESM.tif]

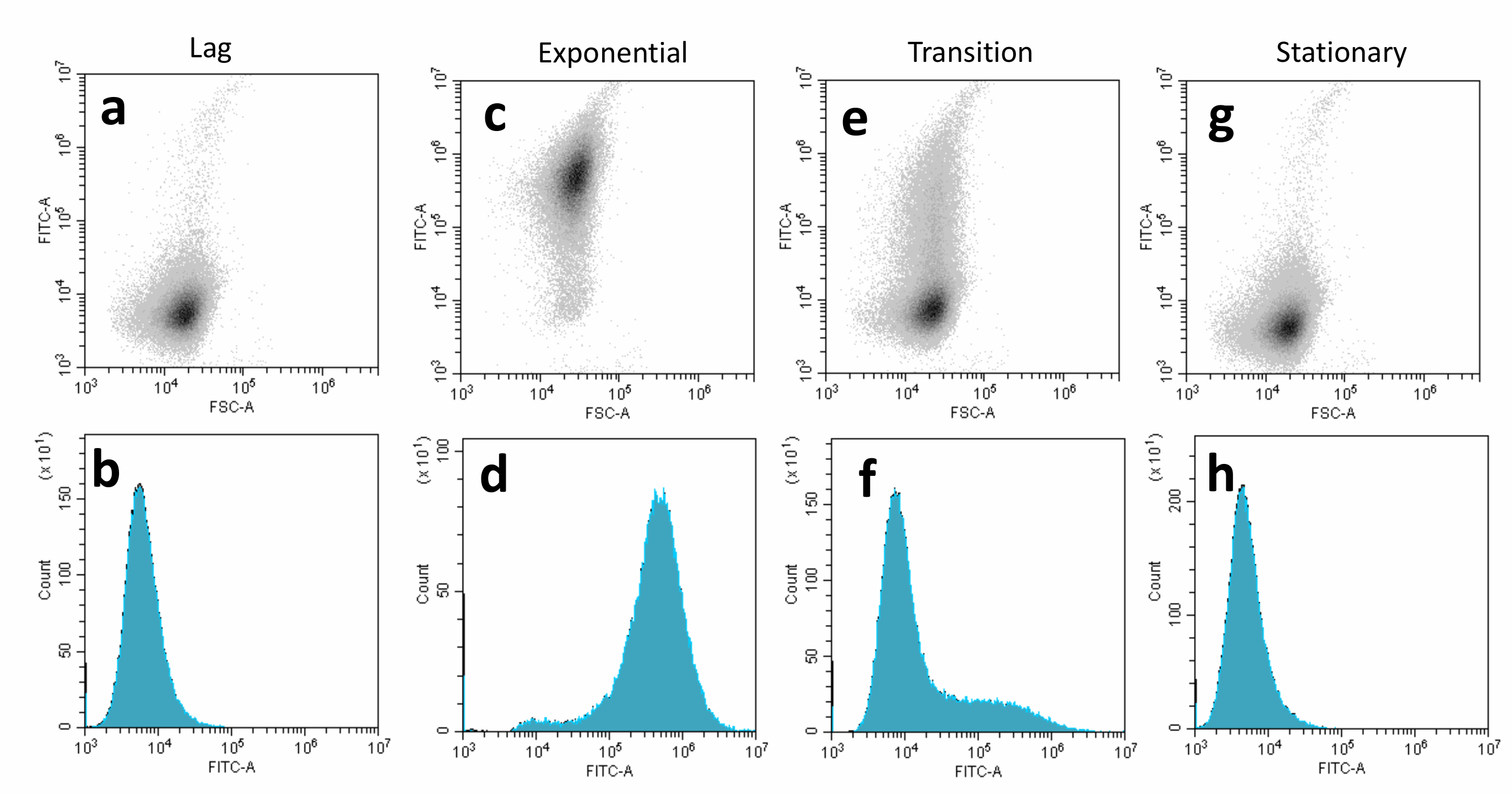

Supplement: Supplementary file 4 — Additional file 4: Figure S4. Flow cytometry analysis of bacterial population with HC-M reporter growing in the minimal medium. Density plot and histogram plot of GFP populations in the lag phase (a,b), exponential phase (c,d), transition between exponential and stationary phases (e,f), and stationary phase (g,h). The BW25113 strain with HC-M ATP reporter was analyzed. Cellular GFP was measured by FITC-A channel. [file 12915_2021_1023_MOESM4_ESM.tif]

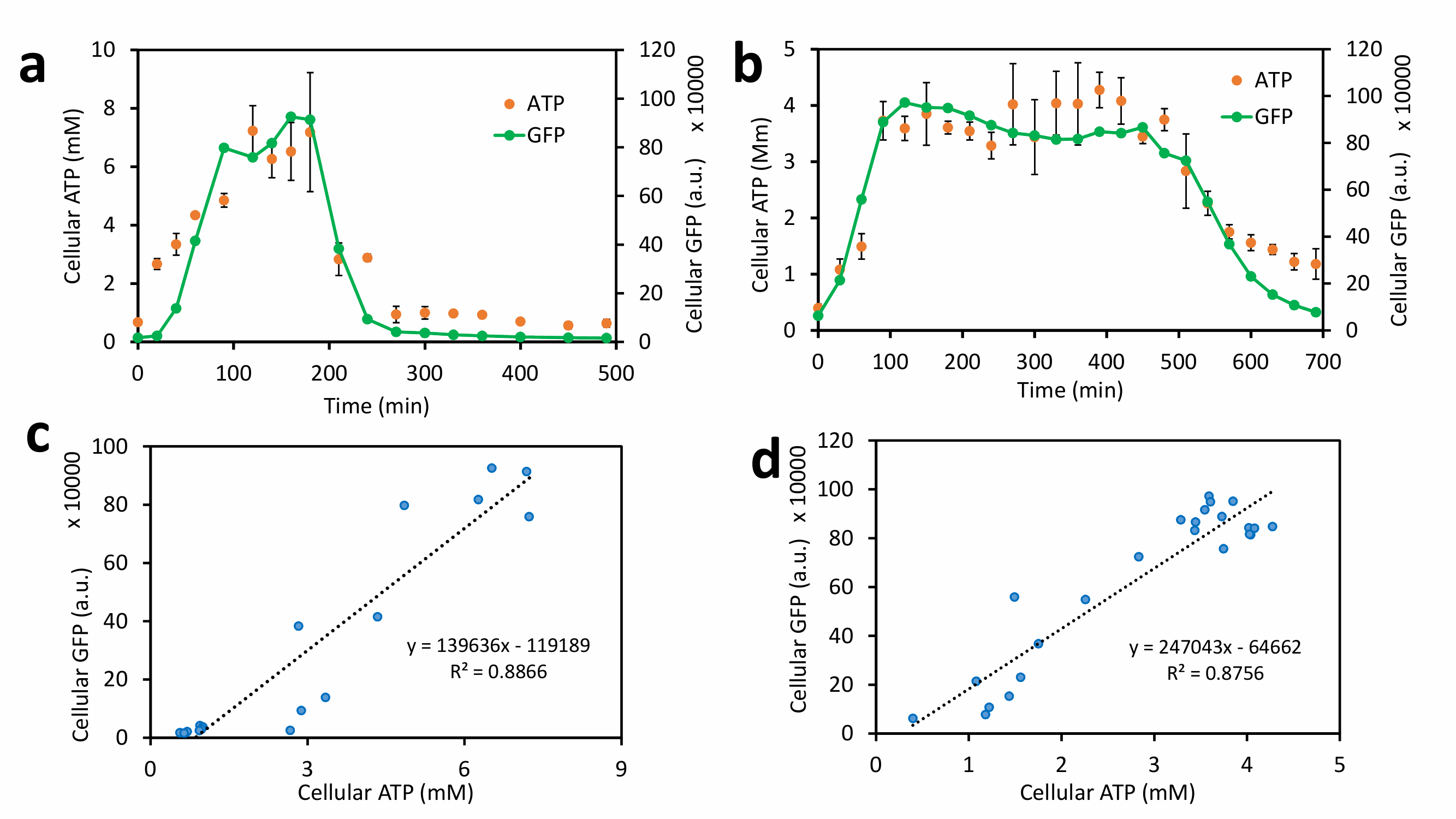

Supplement: Supplementary file 5 — Additional file 5: Figure S5. GFP-ATP correlation analysis of the HC-M reporter in two other strains. (a,b) GFP and ATP dynamics over the growth phases in the JM109DE3 strain in the rich medium (a) and in BL21DE3 strain in the minimal medium (b). ATP was measured by luciferase assay and cellular fluorescence was measured by flow cytometry. Data points are mean values of three independent replicates with one standard deviation (SD). The SD for the GFP signal was relatively small (< 15%) and is thus not shown in the figure. (c,d) Linear correlations between GFP and ATP in the JM109DE3 strain in the rich medium (c) and in BL21DE3 strain in the minimal medium (d). [file 12915_2021_1023_MOESM5_ESM.tif]

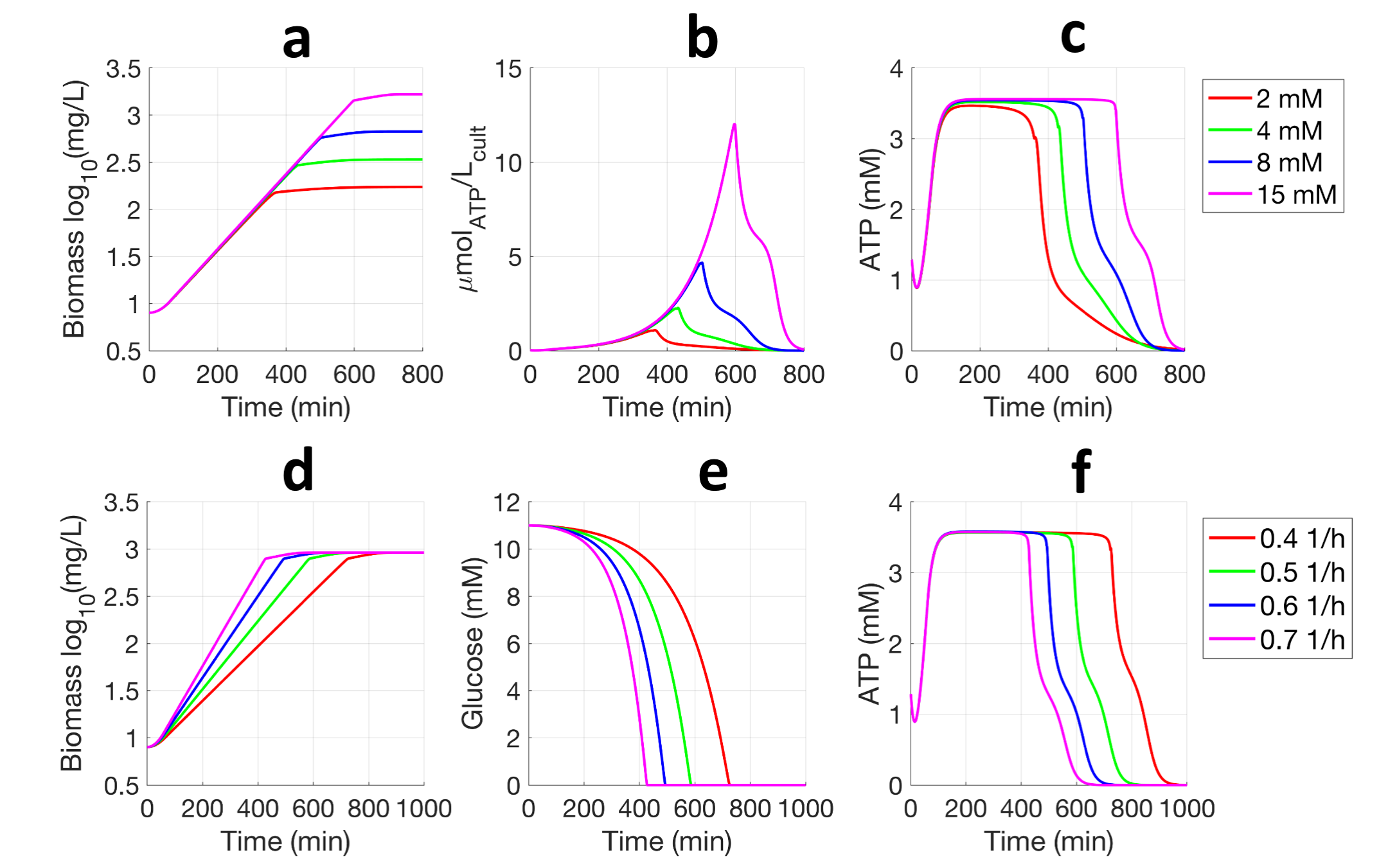

Supplement: Supplementary file 7 — Additional file 7: Figure S7. Sensitivity analysis of the kinetic model by varying initial glucose and growth rate. Dynamics of bacterial growth (a), population ATP (b), and cellular ATP (c) at varying initial glucose concentration. Dynamics of bacterial growth (d), glucose consumption (e), and cellular ATP (f) at varying growth rates. The analysis was performed by varying the initial glucose concentration or growth rate while keeping other parameters identical to those obtained from experiments under the same conditions as those in Fig. 6. Increasing the specific growth rate from 0.4 to 0.7 (1/h) needs the slight adjustment of g from 54.5 to 60.7 (M) for the growth rate sweep. [file 12915_2021_1023_MOESM7_ESM.tiff]

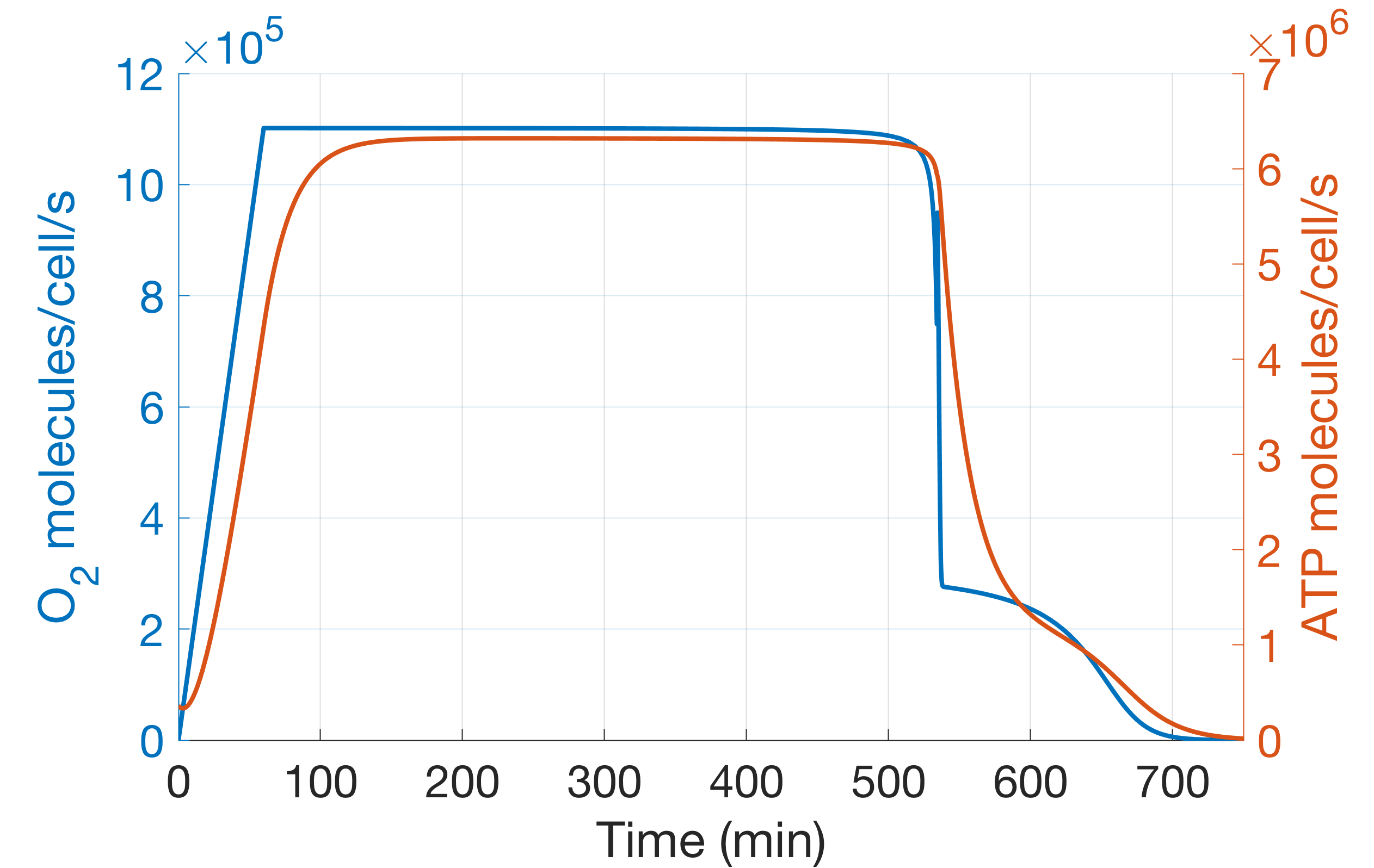

Supplement: Supplementary file 9 — Additional file 9: Figure S8. Comparison of cellular oxygen consumption rate and ATP production rate. Both oxygen flux and ATP production flux were determined from our kinetic model using the experimental data used in Fig. 6. [file 12915_2021_1023_MOESM9_ESM.tif]

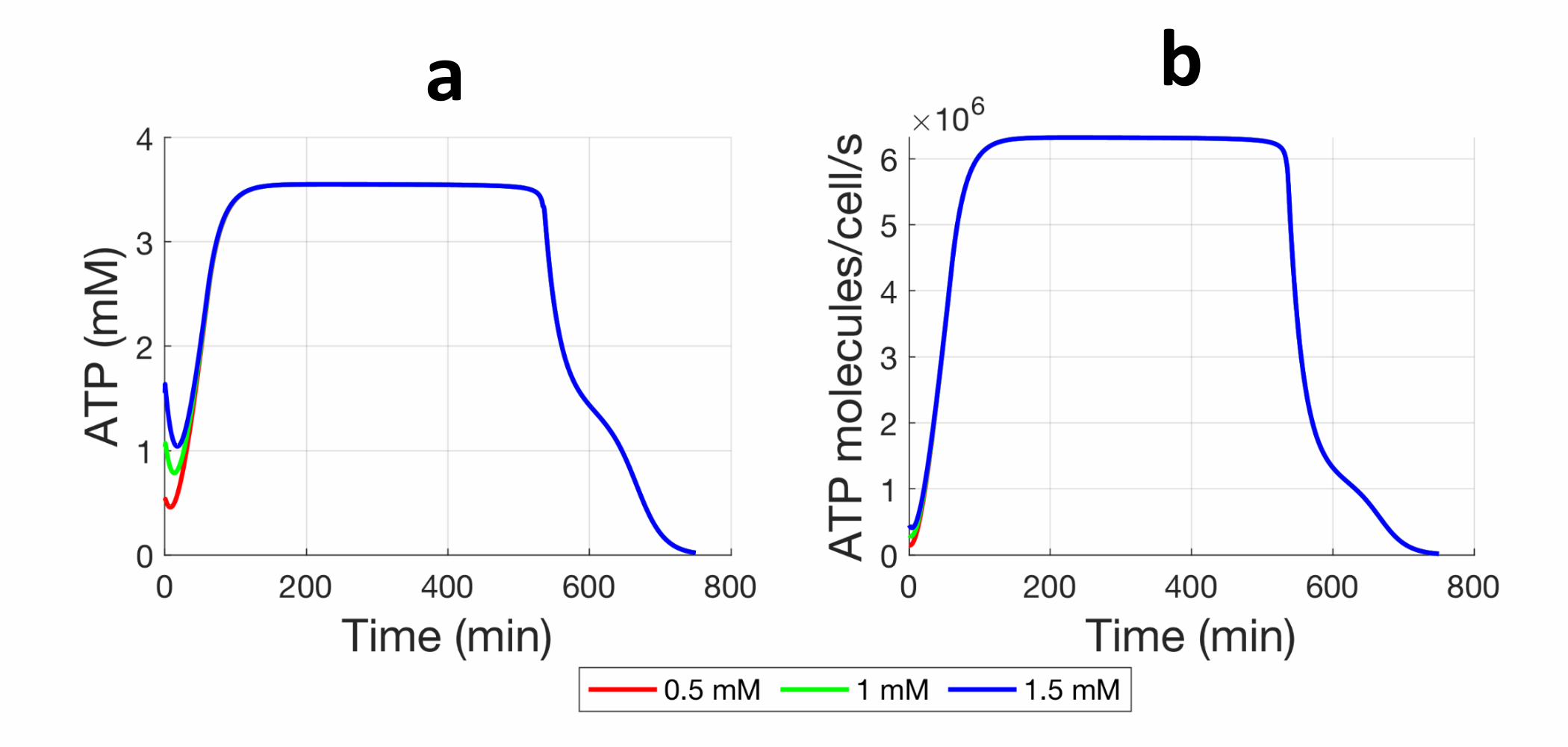

Supplement: Supplementary file 10 — Additional file 10: Figure S9. Model response to varying initial cellular ATP concentrations. All model parameters except the initial cellular ATP fluxes from acetate production and aerobic respiration of glucose were held constant while initial cellular ATP concentration was varied. The initial ATP fluxes change linearly with the initial ATP concentration because we assumed that a cell with a higher initial ATP concentration is in a healthier metabolic state and will initially be producing ATP at a higher rate. [file 12915_2021_1023_MOESM10_ESM.tif]

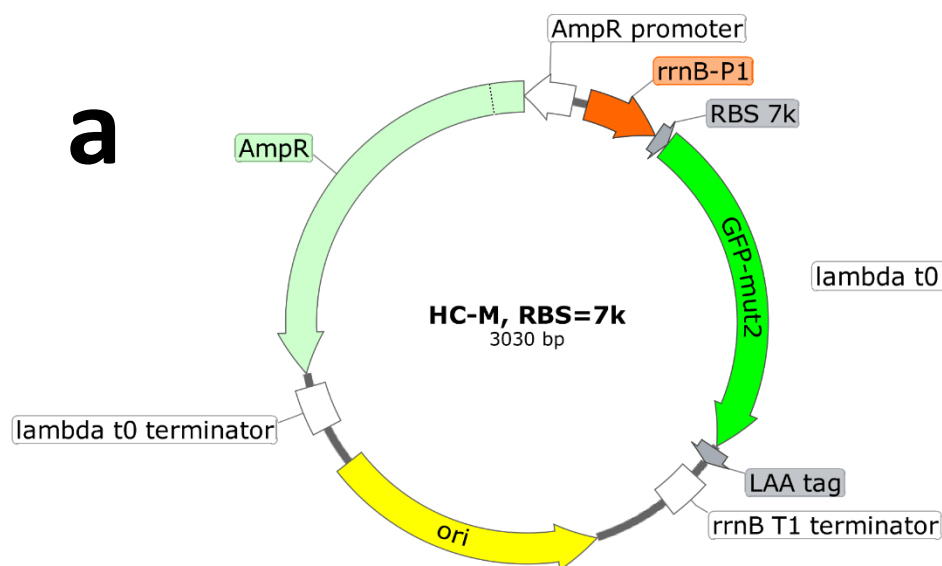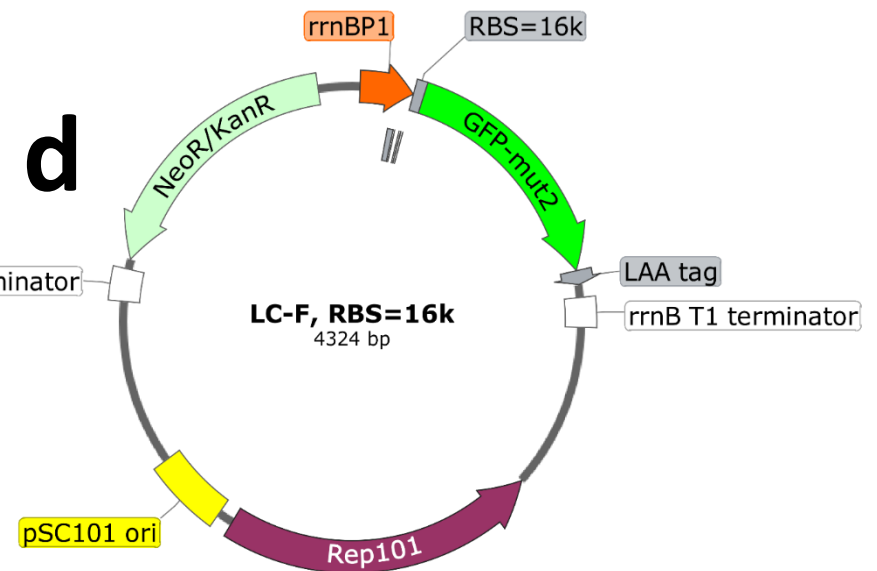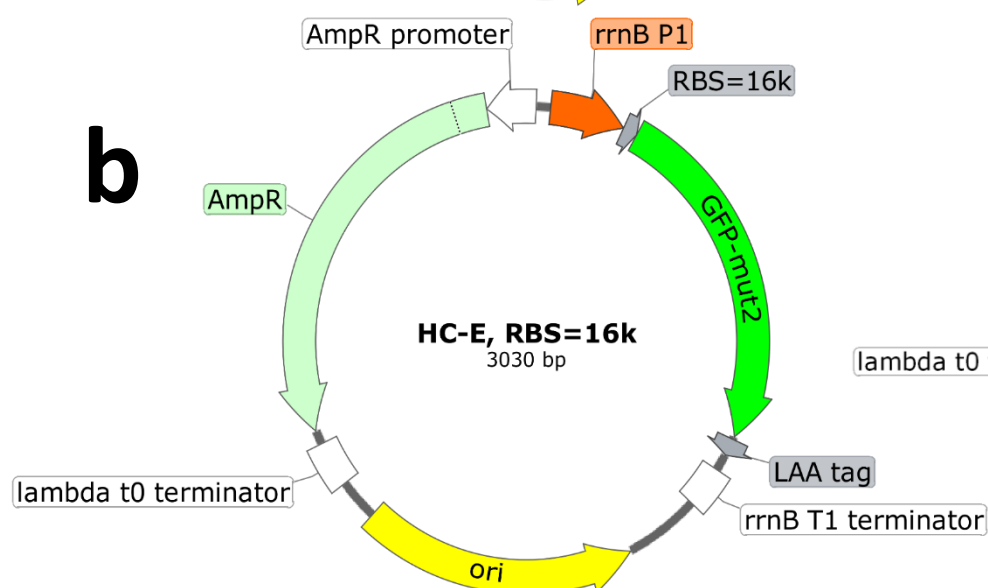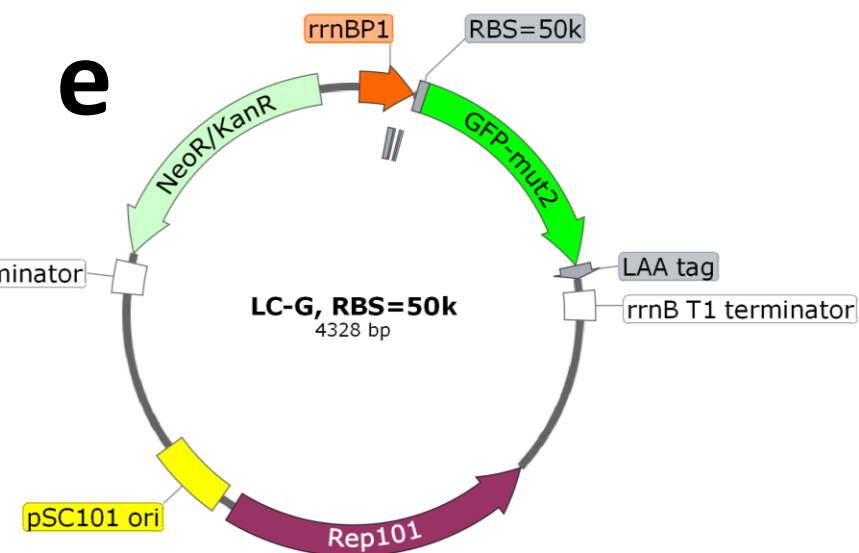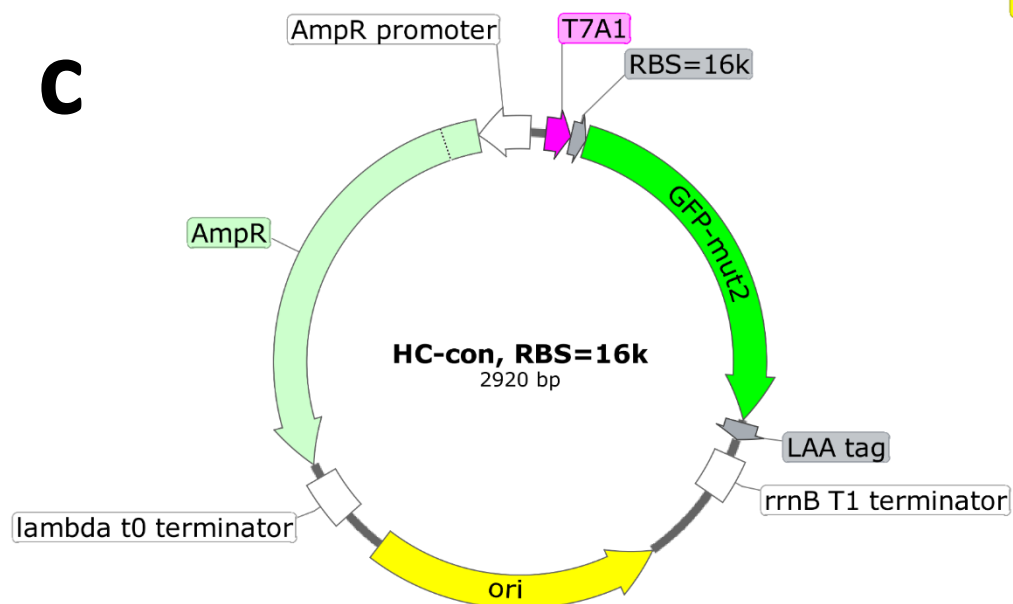

Supplement: Supplementary file 13 — Additional file 13: Figure S10. Plasmids constructed and tested in this work. (a) High-copy-plasmid, low RBS reporter HC-M. (b) High-copy-plasmid, medium RBS reporter HC-E. (c) High-copy-plasmid control reporter HC-con with T7A1 constitutive promoter. (d) Low-copy-plasmid, medium RBS reporter LC-F. (e) Low-copy-plasmid, high RBS reporter LC-G. High-copy plasmids have a ColE1 origin of replication while the low-copy plasmids have a PSC101 origin of replication. [file 12915_2021_1023_MOESM13_ESM.pdf]

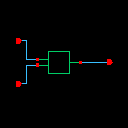

Supplement: Supplementary file 17 — Additional file 17. Cadence files for the kinetic model circuit. The .zip file contains the Cadence library of components (schematics and symbols) and cellview simulation states needed to perform the kinetic model simulations presented in this paper. [file 12915_2021_1023_MOESM17_ESM.zip › rrn_Resubmission/multiplier/schematic/thumbnail_128x128.png]

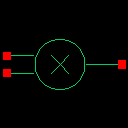

Supplement: Supplementary file 17 — Additional file 17. Cadence files for the kinetic model circuit. The .zip file contains the Cadence library of components (schematics and symbols) and cellview simulation states needed to perform the kinetic model simulations presented in this paper. [file 12915_2021_1023_MOESM17_ESM.zip › rrn_Resubmission/multiplier/symbol/thumbnail_128x128.png]

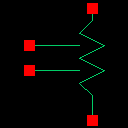

Supplement: Supplementary file 17 — Additional file 17. Cadence files for the kinetic model circuit. The .zip file contains the Cadence library of components (schematics and symbols) and cellview simulation states needed to perform the kinetic model simulations presented in this paper. [file 12915_2021_1023_MOESM17_ESM.zip › rrn_Resubmission/res_MM/symbol/thumbnail_128x128.png]

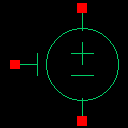

Supplement: Supplementary file 17 — Additional file 17. Cadence files for the kinetic model circuit. The .zip file contains the Cadence library of components (schematics and symbols) and cellview simulation states needed to perform the kinetic model simulations presented in this paper. [file 12915_2021_1023_MOESM17_ESM.zip › rrn_Resubmission/neg_ctrl_vdc/symbol/thumbnail_128x128.png]

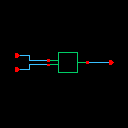

Supplement: Supplementary file 17 — Additional file 17. Cadence files for the kinetic model circuit. The .zip file contains the Cadence library of components (schematics and symbols) and cellview simulation states needed to perform the kinetic model simulations presented in this paper. [file 12915_2021_1023_MOESM17_ESM.zip › rrn_Resubmission/subtractor/schematic/thumbnail_128x128.png]

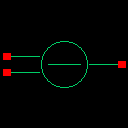

Supplement: Supplementary file 17 — Additional file 17. Cadence files for the kinetic model circuit. The .zip file contains the Cadence library of components (schematics and symbols) and cellview simulation states needed to perform the kinetic model simulations presented in this paper. [file 12915_2021_1023_MOESM17_ESM.zip › rrn_Resubmission/subtractor/symbol/thumbnail_128x128.png]

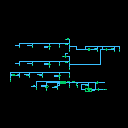

Supplement: Supplementary file 17 — Additional file 17. Cadence files for the kinetic model circuit. The .zip file contains the Cadence library of components (schematics and symbols) and cellview simulation states needed to perform the kinetic model simulations presented in this paper. [file 12915_2021_1023_MOESM17_ESM.zip › rrn_Resubmission/Sarpeskar2021_rrnATP_kinetic_circuit_model/schematic/thumbnail_128x128.png]
