# Supplementary material for: Measuring and modeling energy and power consumption in living microbial cells with a synthetic ATP reporter
Source: BMC Biol. 2021 May 17;19:101. doi: 10.1186/s12915-021-01023-2 (PMC8130387; doi:10.1186/s12915-021-01023-2)
Supplement: Supplementary file 8 — Additional file 8: Table S1. Model parameters for E. coli strain BW25113 grown in minimal medium.The values of KS, tlag and kLa are supported by the references [88–90], respectively. [file 12915_2021_1023_MOESM8_ESM.docx]

**Additional file 8: Table S1** Model parameters for *E. coli* strain BW25113 grown in minimal medium.

|  | **Name** | **Value** | **Units** | **Notes** |
| --- | --- | --- | --- | --- |
| Biomass and Growth | X_0_ | 8 | mg/L | Initial biomass; experimental condition |
|  | μ_max,S_ | 0.55 | h^-1^ | Maximal growth rate on glucose; measured experimentally |
|  | K_S_ | 50 | μM | Monod’s saturation constant for glucose [88] |
|  | μ_max,A_ | 0.099 | h^-1^ | Maximal growth rate on acetate; measured experimentally |
|  | K_A_ | 833 | μM | Monod’s saturation constant for acetate [45] |
|  | S_tran_ | 0.10 | mM | Glucose concentration below which growth on acetate begins [76] |
|  | t_lag_ | 1 | h | lag time [89]; measured experimentally |
|  | m_cell_ | 405 | fg | Cell dry mass [67] |
|  | V_cell_ | 1 | fL | Cell volume [70] |
|  | ρ_cell_ | 405 | g/L | Density of cell; from m_cell_ and V_cell_ |
|  | α | 0.485 | g C/g cell | Stoichiometry in biomass carbon [78] |
| Oxygen | DO_0_ | 206 | μM | Initial dissolved oxygen concentration; measured experimentally |
|  | DO^#^ | 206 | μM | Saturated dissolved oxygen concentration; measured experimentally |
|  | k_L_a | 60 | h^-1^ | Measured experimentally; similar to reported values [90] |
|  | k_occ,S_ | 22.5 | mmol/g | Oxygen consumption rate constant for glucose; fitted; near reported values [45] |
|  | k_occ,A_ | 47.5 | mmol/g | Oxygen consumption rate constant for acetate; fitted; near reported values [85] |
| Glucose | S_0_ | 11 | mM | Initial glucose amount; experimental condition |
| Acetate | A_0_ | 0 | mM | Initial acetate amount; experimental condition |
|  | k_A,pro_ | 7.2 | mmol/g | Fitted; near reported values [85] |
| ATP | ATP_0_ | 1.3 | mM | Initial cellular ATP level; measured experimentally |
|  | I_resp,cell_(t=0) | 300 | uM/s | Initial cellular ATP production rate from glucose respiration; fitted to support ATP_0_ |
|  | I_A,pro,cell_(t=0) | 300 | uM/s | Initial cellular ATP production rate from acetate production; fitted to support ATP_0_ |
|  | τ_delay_ | 20 | min | Delay time to transition ATP fluxes smoothly during growth phase changes |
|  | *m* | 0.450 | s^-1^ | Fitted maintenance energy consumption rate coefficient |
|  | *g* | 58.5 | M | Fitted growth rate-dependent ATP consumption coefficient |
