# Supplementary material for: Measuring and modeling energy and power consumption in living microbial cells with a synthetic ATP reporter
Source: BMC Biol. 2021 May 17;19:101. doi: 10.1186/s12915-021-01023-2 (PMC8130387; doi:10.1186/s12915-021-01023-2)
Supplement: Supplementary file 11 — Additional file 11: Table S2. Calculated ATP values in E. coli BL21(DE3) grown in the minimal medium. a: Power consumption was estimated from 54 kJ/mole ATP [53, 54]. Note: all values are estimated from one biological experiment with three samples measured at each time point. In this experiment, ATP concentration was measured by the HC-M reporter and ATP consumption rates were calculated by the kinetic model. [file 12915_2021_1023_MOESM11_ESM.docx]

**Additional file 11: Table S2** Calculated ATP values in *E. coli* BL21(DE3) grown in the minimal medium

|  | **Lag phase** | **Exponential phase** | **Stationary phase** |
| --- | --- | --- | --- |
| Averaged ATP (mM) | 0.78 | 3.71 | 1.02 |
| ATP consumption rate (million ATP/s/cell) | 1.06 | 8.97 | 1.65 |
| Power consumption (pW)a | 0.095 | 0.81 | 0.015 |
| ATP turnover time (s) | 0.45 | 0.25 | 0.36 |

a: Power consumption was estimated from 54 kJ/mole ATP [53, 54]. Note: all values are estimated from one biological experiment with three samples measured at each time point. In this experiment, ATP concentration was measured by the HC-M reporter and ATP consumption rates were calculated by the kinetic model.
