# Supplementary material for: Measuring and modeling energy and power consumption in living microbial cells with a synthetic ATP reporter
Source: BMC Biol. 2021 May 17;19:101. doi: 10.1186/s12915-021-01023-2 (PMC8130387; doi:10.1186/s12915-021-01023-2)
Supplement: Supplementary file 12 — Additional file 12: Table S3. Model parameters of the model for E. coli strain BL21(DE3) grown in minimal medium. These parameters are used in the simulation that calculates the dynamic ATP values in Additional file 11: Table S2. These parameters are same to those used for the BW25113 strain with only slight changes for a few parameters to account for strain-to-strain variations. [file 12915_2021_1023_MOESM12_ESM.docx]

**Additional file 12: Table S3** Model parameters for *E. coli* strain BL21(DE3) grown in minimal medium.

|  | **Name** | **Value** | **Units** | **Notes** |
| --- | --- | --- | --- | --- |
| Biomass and Growth | X_0_ | 5 | mg/L | Initial biomass; experimental condition |
|  | μ_max,S_ | 0.606 | h^-1^ | Maximal growth rate on glucose; measured experimentally |
|  | K_S_ | 50 | μM | Monod’s saturation constant for glucose [88] |
|  | μ_max,A_ | 0.156 | h^-1^ | Maximal growth rate on acetate; measured experimentally |
|  | K_A_ | 833 | μM | Monod’s saturation constant for acetate [45] |
|  | S_tran_ | 0.10 | mM | Glucose concentration below which growth on acetate begins [76] |
|  | t_lag_ | 0.25 | h | lag time [89]; measured experimentally |
|  | m_cell_ | 405 | fg | Cell dry mass [67] |
|  | V_cell_ | 1 | fL | Cell volume [70] |
|  | ρ_cell_ | 405 | g/L | Density of cell; from m_cell_ and V_cell_ |
|  | α | 0.485 | g C/g cell | Stoichiometry in biomass carbon [78] |
| Oxygen | DO_0_ | 206 | μM | Initial dissolved oxygen concentration; measured experimentally |
|  | DO^#^ | 206 | μM | Saturated dissolved oxygen concentration; measured experimentally |
|  | k_L_a | 60 | h^-1^ | Measured experimentally; similar to reported values [90] |
|  | k_occ,S_ | 39.5 | mmol/g | Oxygen consumption rate constant for glucose; fitted; near reported values [45] |
|  | k_occ,A_ | 47.5 | mmol/g | Oxygen consumption rate constant for acetate; fitted; near reported values [85] |
| Glucose | S_0_ | 12 | mM | Initial glucose amount; experimental condition |
| Acetate | A_0_ | 0 | mM | Initial acetate amount; experimental condition |
|  | k_A,pro_ | 1.3 | mmol/g | Fitted; near reported values [85] |
| ATP | ATP_0_ | 0.4 | mM | Initial cellular ATP level; measured experimentally |
|  | I_resp,cell_(t=0) | 100 | uM/s | Initial cellular ATP production rate from glucose respiration; fitted to support ATP_0_ |
|  | I_A,pro,cell_(t=0) | 100 | uM/s | Initial cellular ATP production rate from acetate production; fitted to support ATP_0_ |
|  | τ_delay_ | 20 | min | Delay time to transition ATP fluxes smoothly during growth phase changes |
|  | *m* | 0.450 | s^-1^ | Fitted maintenance energy consumption rate coefficient |
|  | *g* | 78.5 | M | Fitted growth rate-dependent ATP consumption coefficient |
