# Supplementary material for: Measuring and modeling energy and power consumption in living microbial cells with a synthetic ATP reporter
Source: BMC Biol. 2021 May 17;19:101. doi: 10.1186/s12915-021-01023-2 (PMC8130387; doi:10.1186/s12915-021-01023-2)
Supplement: Supplementary file 14 — Additional file 14. The inserted DNA sequences of the plasmids used in this study. [file 12915_2021_1023_MOESM14_ESM.docx]

**DNA sequences of the plasmids made in this study**

Only the inserted region sequences are shown below and plasmid backbone sequences are omitted.

Yellow: *rrnB* P1 promoter; Cyan: RBS; Green; GFP-mut2; Gray: fast degradation tag; pink: T7A1 promoter.

1. **HC-M**

GccaggagctgaacaattattgcccgttttacagcgttacggcttcgaaacgctcgaaaaactggcagttttaggctgatttggttgaatgttgcgcggtcagaaaattattttaaatttcctcttgtcaggccggaataactccctataatgcgccaccAAACATTCACCGATATCCGAACGGAGGTAGTTATGTCGAAAGGTGAGGAGTTATTCACAGGGGTCGTGCCCATTTTAGTGGAACTTGACGGAGATGTCAACGGTCACAAGTTTAGTGTGTCTGGCGAGGGGGAGGGGGATGCCACGTATGGCAAGTTGACCTTGAAGTTTATCTGTACTACCGGAAAATTACCCGTGCCGTGGCCGACATTGGTCACAACTTTTGCTTACGGTCTTCAGTGCTTCGCTCGCTACCCCGATCATATGAAGCAGCATGATTTTTTCAAAAGCGCGATGCCCGAGGGGTATGTTCAAGAACGTACCATTTTCTTCAAGGACGATGGCAACTACAAGACTCGTGCAGAGGTCAAATTTGAGGGTGACACTCTTGTAAATCGCATTGAGTTAAAGGGTATCGATTTCAAAGAAGACGGCAATATTCTTGGGCACAAATTAGAATACAACTACAACAGCCATAACGTGTATATTATGGCTGACAAACAAAAGAATGGAATTAAGGTTAATTTTAAGATCCGCCATAATATTGAGGACGGATCAGTTCAGCTGGCGGATCACTATCAACAGAATACACCGATCGGTGACGGCCCTGTGCTGCTTCCAGACAACCACTATTTATCCACTCAGTCCAAGTTATCTAAAGACCCCAATGAAAAACGCGATCATATGGTTTTGTTAGAGTTCGTAACGGCTGCAGGAATCACTCATGGTATGGACGAGTTGTATAAAAGGCCTGCAGCAAACGACGAAAACTACGCTTTAGCAGCTTAA

1. **HC-E**

GccaggagctgaacaattattgcccgttttacagcgttacggcttcgaaacgctcgaaaaactggcagttttaggctgatttggttgaatgttgcgcggtcagaaaattattttaaatttcctcttgtcaggccggaataactccctataatgcgccaccAAGAATTCACCGATATCCGAACGGAGGTAGTTATGTCGAAAGGTGAGGAGTTATTCACAGGGGTCGTGCCCATTTTAGTGGAACTTGACGGAGATGTCAACGGTCACAAGTTTAGTGTGTCTGGCGAGGGGGAGGGGGATGCCACGTATGGCAAGTTGACCTTGAAGTTTATCTGTACTACCGGAAAATTACCCGTGCCGTGGCCGACATTGGTCACAACTTTTGCTTACGGTCTTCAGTGCTTCGCTCGCTACCCCGATCATATGAAGCAGCATGATTTTTTCAAAAGCGCGATGCCCGAGGGGTATGTTCAAGAACGTACCATTTTCTTCAAGGACGATGGCAACTACAAGACTCGTGCAGAGGTCAAATTTGAGGGTGACACTCTTGTAAATCGCATTGAGTTAAAGGGTATCGATTTCAAAGAAGACGGCAATATTCTTGGGCACAAATTAGAATACAACTACAACAGCCATAACGTGTATATTATGGCTGACAAACAAAAGAATGGAATTAAGGTTAATTTTAAGATCCGCCATAATATTGAGGACGGATCAGTTCAGCTGGCGGATCACTATCAACAGAATACACCGATCGGTGACGGCCCTGTGCTGCTTCCAGACAACCACTATTTATCCACTCAGTCCAAGTTATCTAAAGACCCCAATGAAAAACGCGATCATATGGTTTTGTTAGAGTTCGTAACGGCTGCAGGAATCACTCATGGTATGGACGAGTTGTATAAAAGGCCTGCAGCAAACGACGAAAACTACGCTTTAGCAGCTTAA

1. **LC-F** (sequences same as HC-E)

GccaggagctgaacaattattgcccgttttacagcgttacggcttcgaaacgctcgaaaaactggcagttttaggctgatttggttgaatgttgcgcggtcagaaaattattttaaatttcctcttgtcaggccggaataactccctataatgcgccaccAAAGAATTCACCGATATCCGAACGGAGGTAGTTATGTCGAAAGGTGAGGAGTTATTCACAGGGGTCGTGCCCATTTTAGTGGAACTTGACGGAGATGTCAACGGTCACAAGTTTAGTGTGTCTGGCGAGGGGGAGGGGGATGCCACGTATGGCAAGTTGACCTTGAAGTTTATCTGTACTACCGGAAAATTACCCGTGCCGTGGCCGACATTGGTCACAACTTTTGCTTACGGTCTTCAGTGCTTCGCTCGCTACCCCGATCATATGAAGCAGCATGATTTTTTCAAAAGCGCGATGCCCGAGGGGTATGTTCAAGAACGTACCATTTTCTTCAAGGACGATGGCAACTACAAGACTCGTGCAGAGGTCAAATTTGAGGGTGACACTCTTGTAAATCGCATTGAGTTAAAGGGTATCGATTTCAAAGAAGACGGCAATATTCTTGGGCACAAATTAGAATACAACTACAACAGCCATAACGTGTATATTATGGCTGACAAACAAAAGAATGGAATTAAGGTTAATTTTAAGATCCGCCATAATATTGAGGACGGATCAGTTCAGCTGGCGGATCACTATCAACAGAATACACCGATCGGTGACGGCCCTGTGCTGCTTCCAGACAACCACTATTTATCCACTCAGTCCAAGTTATCTAAAGACCCCAATGAAAAACGCGATCATATGGTTTTGTTAGAGTTCGTAACGGCTGCAGGAATCACTCATGGTATGGACGAGTTGTATAAAAGGCCTGCAGCAAACGACGAAAACTACGCTTTAGCAGCTTAA

1. **LC-G**

GccaggagctgaacaattattgcccgttttacagcgttacggcttcgaaacgctcgaaaaactggcagttttaggctgatttggttgaatgttgcgcggtcagaaaattattttaaatttcctcttgtcaggccggaataactccctataatgcgccaccAAGAATTCCGGAGCAGCCAACAGGGGGAGGTTAATCATGTCGAAAGGTGAGGAGTTATTCACAGGGGTCGTGCCCATTTTAGTGGAACTTGACGGAGATGTCAACGGTCACAAGTTTAGTGTGTCTGGCGAGGGGGAGGGGGATGCCACGTATGGCAAGTTGACCTTGAAGTTTATCTGTACTACCGGAAAATTACCCGTGCCGTGGCCGACATTGGTCACAACTTTTGCTTACGGTCTTCAGTGCTTCGCTCGCTACCCCGATCATATGAAGCAGCATGATTTTTTCAAAAGCGCGATGCCCGAGGGGTATGTTCAAGAACGTACCATTTTCTTCAAGGACGATGGCAACTACAAGACTCGTGCAGAGGTCAAATTTGAGGGTGACACTCTTGTAAATCGCATTGAGTTAAAGGGTATCGATTTCAAAGAAGACGGCAATATTCTTGGGCACAAATTAGAATACAACTACAACAGCCATAACGTGTATATTATGGCTGACAAACAAAAGAATGGAATTAAGGTTAATTTTAAGATCCGCCATAATATTGAGGACGGATCAGTTCAGCTGGCGGATCACTATCAACAGAATACACCGATCGGTGACGGCCCTGTGCTGCTTCCAGACAACCACTATTTATCCACTCAGTCCAAGTTATCTAAAGACCCCAATGAAAAACGCGATCATATGGTTTTGTTAGAGTTCGTAACGGCTGCAGGAATCACTCATGGTATGGACGAGTTGTATAAAAGGCCTGCAGCAAACGACGAAAACTACGCTTTAGCAGCTTAA

1. **HC-Con**

TTATCAAAAAGAGTATTGACTTAAAGTCTAACCTATAGGATACTTACAGCCAGAATTCACCGATATCCGAACGGAGGTAGTTATGTCGAAAGGTGAGGAGTTATTCACAGGGGTCGTGCCCATTTTAGTGGAACTTGACGGAGATGTCAACGGTCACAAGTTTAGTGTGTCTGGCGAGGGGGAGGGGGATGCCACGTATGGCAAGTTGACCTTGAAGTTTATCTGTACTACCGGAAAATTACCCGTGCCGTGGCCGACATTGGTCACAACTTTTGCTTACGGTCTTCAGTGCTTCGCTCGCTACCCCGATCATATGAAGCAGCATGATTTTTTCAAAAGCGCGATGCCCGAGGGGTATGTTCAAGAACGTACCATTTTCTTCAAGGACGATGGCAACTACAAGACTCGTGCAGAGGTCAAATTTGAGGGTGACACTCTTGTAAATCGCATTGAGTTAAAGGGTATCGATTTCAAAGAAGACGGCAATATTCTTGGGCACAAATTAGAATACAACTACAACAGCCATAACGTGTATATTATGGCTGACAAACAAAAGAATGGAATTAAGGTTAATTTTAAGATCCGCCATAATATTGAGGACGGATCAGTTCAGCTGGCGGATCACTATCAACAGAATACACCGATCGGTGACGGCCCTGTGCTGCTTCCAGACAACCACTATTTATCCACTCAGTCCAAGTTATCTAAAGACCCCAATGAAAAACGCGATCATATGGTTTTGTTAGAGTTCGTAACGGCTGCAGGAATCACTCATGGTATGGACGAGTTGTATAAAAGGCCTGCAGCAAACGACGAAAACTACGCTTTAGCAGCTTAA
